# Trimerisation of carbon suboxide at a di-titanium centre to form a pyrone ring system†
†Dedicated to the memory of Prof. Greg Hillhouse – a pioneer in exploring the reactivity of C_3_O_2_ with transition metal complexes.
‡
‡Electronic supplementary information (ESI) available: Experimental and synthetic procedures, characterisation data, additional NMR and IR spectroscopic data, computational details and Cartesian coordinates for all computed molecular structures, and crystallographic methods employed in this work. CCDC 1817210 and 1817211. For ESI and crystallographic data in CIF or other electronic format see DOI: 10.1039/c8sc01127c


**DOI:** 10.1039/c8sc01127c

**Published:** 2018-05-08

**Authors:** Nikolaos Tsoureas, Jennifer C. Green, F. Geoffrey N. Cloke, Horst Puschmann, S. Mark Roe, Graham Tizzard

**Affiliations:** a School of Life Sciences , Department of Chemistry , University of Sussex , Falmer , Brighton , BN1 9QJ , UK . Email: F.G.Cloke@sussex.ac.uk; b Department of Chemistry , University of Oxford , Inorganic Chemistry Laboratory , South Parks Road , Oxford OX1 3QR , UK; c OlexSys Ltd , Chemistry Department , Durham University , DH1 3LE , UK; d EPSRC National Crystallography Service , School of Chemistry , University of Southampton , Highfield Campus , Southampton SO17 1BJ , UK

## Abstract

The reaction of the *syn*-bimetallic bis(pentalene)dititanium complex Ti_2_(μ:η^5^,η^5^-Pn^†^)_2_ (Pn^†^ = C_8_H_4_(1,4-Si^i^Pr_3_)_2_) **1** with carbon suboxide (O=C=C=C=O, C_3_O_2_) results in trimerisation of the latter and formation of the structurally characterised complex [{Ti_2_(μ:η^5^,η^5^-Pn^†^)_2_}{μ-C_9_O_6_}]. The trimeric bridging C_9_O_6_ unit in the latter contains a 4-pyrone core, a key feature of both the hexamer and octamer of carbon suboxide which are formed in the body from trace amounts of C_3_O_2_ and are, for example, potent inhibitors of Na^+^/K^+^-ATP-ase. The mechanism of this reaction has been studied in detail by DFT computational studies, which also suggest that the reaction proceeds *via* the initial formation of a mono-adduct of **1** with C_3_O_2_. Indeed, the carefully controlled reaction of **1** with C_3_O_2_ affords [Ti_2_(μ:η^5^,η^5^-Pn^†^)_2_ (η^2^-C_3_O_2_)], as the first structurally authenticated complex of carbon suboxide.

## Abstract 2

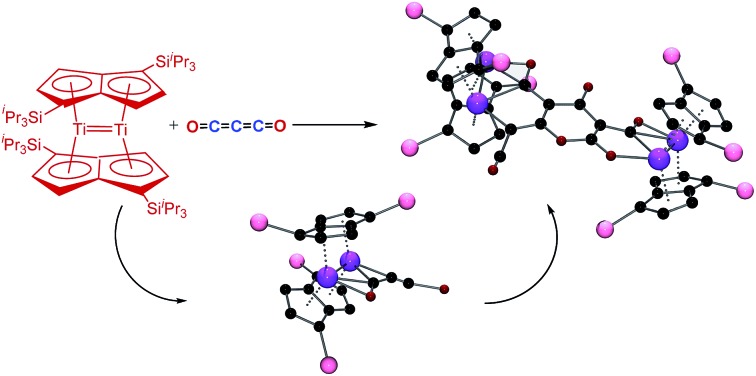
Bis(pentalene)dititanium Ti_2_(μ:η^5^,η^5^-Pn^†^)_2_ trimerises carbon suboxide (O=C=C=C=O) to form [{Ti_2_(μ:η^5^,η^5^-Pn^†^)_2_}{μ-C_9_O_6_}], which contains a 4-pyrone core, *via* the monoadduct [Ti_2_(μ:η^5^,η^5^-Pn^†^)_2_ (η^2^-C_3_O_2_)].

## Introduction

Unlike the plethora of catalytic and stoichiometric transformations of carbon's most common oxides (*i.e.* CO and CO_2_) promoted by well-defined molecular complexes,^1^–^4^ there is a disproportionate lack of examples featuring the activation and subsequent transformation of the sub-oxides of carbon. C_3_O_2_ is the first in the series of the synthetically available carbon sub-oxides featuring an odd number of carbons^5^,^6^ (predicted to augment their stability^7^), and its spectroscopic^8^ and physical properties^9^,^10^ have been extensively studied. Its molecular structure in the solid state has been reported and shows a linear structure,^11^ whereas in the gas phase computational^12^ and spectroscopic studies^13^ confirm a bent structure with a bond angle of 156°. Similarly, aspects of its reactivity with a variety of organic substrates^14^ (*e.g.* ylides^15^–^17^) and main-group^18^ bonds have been reported since its first synthesis. Although C_3_O_2_ (hereafter referred to as carbon suboxide) is relatively unstable (it auto-polymerises above 0 °C but can be stored indefinitely below –35 °C), it is moderately straightforward to prepare *via* the dehydration of malonic esters^5^ or malonic acid^19^ with phosphorus pentoxide. The polymer produced by its self-polymerisation has a band-like structure with condensed α-pyrone rings and has been studied for its electronic properties.^20^,^21^

**Fig. 1:**
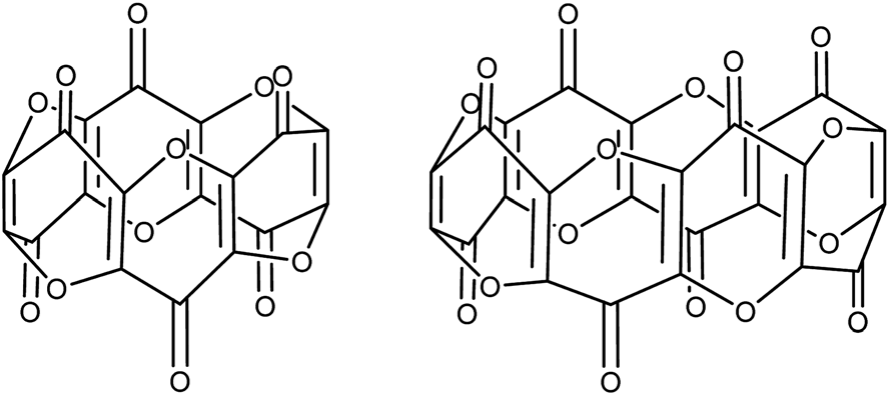
Hexamers (left) and octamers (right) of carbon suboxide relevant to biological processes.

Carbon suboxide is also formed in small quantities *in vivo* during biochemical processes that normally produce carbon monoxide, for example, during heme oxidation by heme oxygenase-1 (HO-1). It is then rapidly oligomerised into macrocyclic structures, predominantly cyclic hexamers and octamers (Fig. 1), which contain fused 4-pyrone rings and are potent inhibitors of Na^+^/K^+^-ATP-ase and Ca-dependent ATP-ase; larger carbon suboxide based macrocycles are proposed to be natriuretic and endogenous digitalis like factors (EDLFs).^22^–^24^

In terms of coordination chemistry, it was proposed that the thermal decomposition of Ag_3_C_3_O_2_ to produce C_3_O_2_ involved a coordination complex of Ag,^25^ and subsequent studies of the reactivity of C_3_O_2_ towards Pt(0), Pt(ii) and Rh(i) complexes by Pandolfo *et al.* proposed the formation of C_3_O_2_ complexes but lack of structural data plagued these early investigations.^26^ Nevertheless, later studies from the same group^27^,^28^ as well as that of Hillhouse^29^ demonstrated some aspects of the reactivity of C_3_O_2_ with organometallic fragments by isolating, for example, the products of its insertion into M–H bonds. A main problem of these early studies was the propensity of C_3_O_2_ to act as a source of ketene (:C=C=O) and CO. Thus, in the presence of phosphorous containing ligands in the coordination sphere of the metal centre, this led to the formation of the corresponding phosphorous-ylides, as shown by Hillhouse *et al.* by the reaction of C_3_O_2_ with WCl_2_(PMePh_2_)_4_ furnishing WCl_2_(CO)(PMePh_2_)_2_{C,C′:η^2^-C(O)CPMePh_2_}.^30^ List and Hillhouse further showed that C_3_O_2_ can displace COD (COD = 1,5-cyclo-octadiene) in (PPh_3_)_2_Ni(COD) to yield (PPh_3_)_2_Ni{C,C′:η^2^-C_3_O_2_} where the C_3_O_2_ ligand coordinates *via* the central and one of the terminal carbons,^31^ although this could not be confirmed crystallographically. Gas phase spectroscopic^32^ and theoretical^33^ studies on the bonding of C_3_O_2_ to late transition metal centres have also been reported, but, and to the best of our knowledge, there have been no other reports investigating the interaction of C_3_O_2_ with organometallic or other coordination compounds. Undoubtedly one of the reasons is its capricious nature, which has favored *in silico* studies of its reactivity especially towards transition metals.^34^,^35^ Indeed, C_3_O_2_ is one of the least explored ‘small molecules’ from a synthetic chemist's point of view, a fact underlined by only two short reviews in the current literature.^14^,^36^

**Fig. 2:**
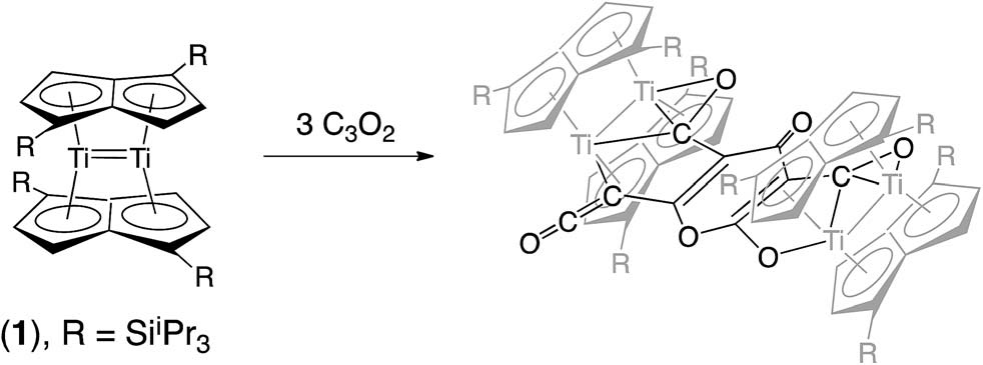
Trimerization of C_3_O_2_ by [Ti_2_(μ:η^5^,η^5^-Pn^†^)_2_].

We have previously reported on the synthesis,^37^ and diverse reactivity^38^–^41^ of the *syn*-bimetallic complex [Ti_2_(μ:η^5^,η^5^-Pn^†^)_2_] (Pn^†^ = C_8_H_4_(Si^i^Pr_3_)_2_) (**1**) towards CO, CO_2_, and heteroallenes and therefore envisioned that (**1**) might be a good candidate for the binding and activation of C_3_O_2_. Herein we present the unprecedented trimerization of C_3_O_2_ promoted by (**1**), Fig. 2, as well as experimental and computational investigations into the mechanism of this reaction.

## Results and discussion

**Fig. 3:**
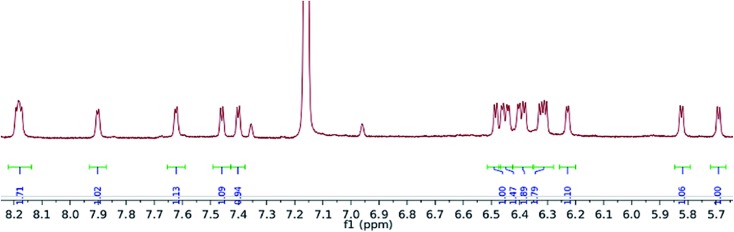
Aromatic region of ^1^H NMR spectrum of (**2**) (C_6_D_6_).

Exposure of a crimson-red toluene solution of (**1**) to C_3_O_2_ at –78 °C, instantly produced a homogeneous brown solution which, upon warming to –35 °C and then slowly to room temperature, deposited some C_3_O_2_ polymer, together with a brown supernatant. Filtration of the reaction mixture and work up of the filtrate afforded a brown-green solid, which was isolated in moderate to good yields (yields are dependent on the final temperature of the solution and vary between 40 and 66%), and proved to be a diamagnetic, spectroscopically pure new compound (**2**). The ^1^H-NMR spectrum of (Fig. 3) (**2**) consisted of 16 doublets in the aromatic region signifying the formation of a dimer exhibiting four inequivalent pentalene environments; this was further substantiated by the observation of eight peaks in the ^29^Si{^1^H}-NMR spectrum of (**2**).

**Fig. 4:**
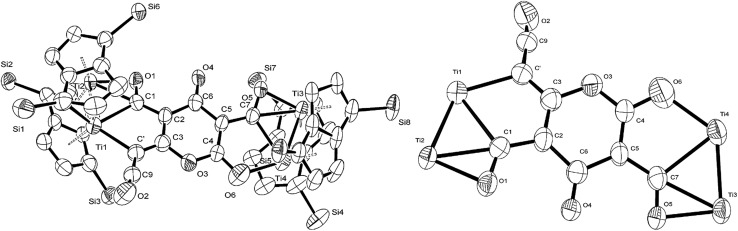
Molecular structure of (**2**) (left-H atoms and ^i^Pr groups removed for clarity) as well as its core (right-with pentalene ligand scaffold removed for extra clarity) showing 50% probability ellipsoids. Selected bond lengths (Å) and angles (°): Ti1–Ti2: 2.4648(14), Ti3–Ti4: 2.4834(16), Ti2–C1: 2.17(3), Ti2–O1: 2.13(3), Ti1–C1: 2.41(2), Ti1–C′: 2.32(2), Ti4–O6: 2.10(2), Ti3–C7: 2.316(17), Ti3–O5: 2.133(19), Ti4–C7: 2.476(17), C′–C9: 1.40(3), C9–O2: 1.161(17), C1–O1: 1.30(3), C7–O5: 1.28(3), C′–C9–O2: 172.4(16), C3–C′–Ti1: 114.9(16), Ti1–C1–Ti2: 64.9(7), C1–O1–Ti2: 74.1(16), C1–Ti2–O1: 35.3(8), C1–Ti1–C′: 75.0(8), O1–Ti2–Ti1: 97.5(6), Ti1–C1–Ti2: 64.9(7), C2–C1–Ti2: 171.1(19), O6–Ti4–Ti3: 131.0(5), C4–O6–Ti4: 115.7(13), Ti3–C7–O5: 65.6(11), C7–Ti3–O5: 33.1(7), Ti3–O5–C7: 81.3(12), C5–C7–Ti3: 169.5(13), Ti3–C7–Ti4: 62.3(4), O6–Ti4–C7: 75.4(7), O5–Ti3–Ti4: 95.1(5), O5–C7–C5: 122.5(17).

**Fig. 5:**
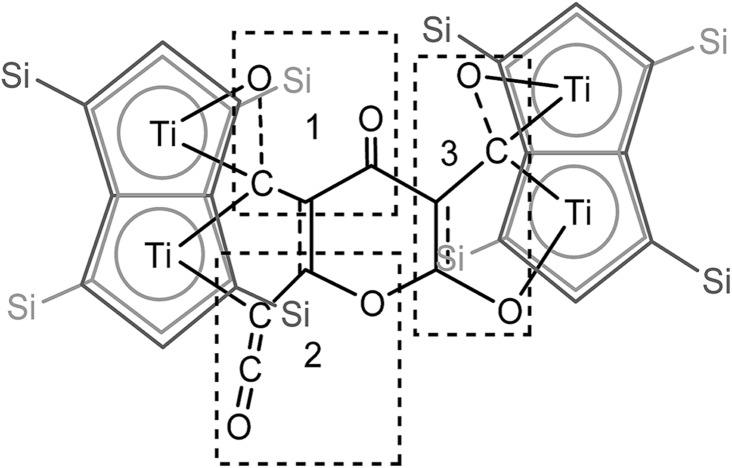
Representation of the molecular structure of (**2**), highlighting the origin of the [C_9_O_6_] core in (**2**).

The ^13^C{^1^H}-NMR spectrum of (**2**) displayed 41 resonance in the region between 389–96 ppm, 32 of which were assigned to the four inequivalent pentalene environments (our empirical observation is that resonances associated with this type of pentalene ligand scaffold in the μ:η^5^,η^5^ coordination geometry appear in the region between 90 and 145 ppm (ref. 38–41)), with the nine remaining signals found in the downfield part of the spectrum (150–400 ppm) and which corresponded to quaternary carbons. At this point, it is interesting to note that such high field resonances (300–400 ppm region) in ^13^C-NMR spectra have been observed for complexes of Zr(iv) and Th(iv) featuring dihaptoacyl ligands with substantial oxy-carbene character.^42^,^43^ The IR spectrum (thin film) of (**2**) showed a strong absorption at 2061 cm^–1^ characteristic of a C=C=O moiety along with bands at 1658, 1591 and 1532 cm^–1^ characteristic of carbonyl functionalities, but also in agreement with haptoacyl ligands with a strong oxo-carbene character.^42^,^43^ An X-ray diffraction study revealed the molecular structure of this new complex (**2**) (Fig. 4), which is consistent with the solution NMR data discussed above, and unequivocally demonstrates the first example of the trimerisation of C_3_O_2_ (Fig. 5). At this point, it is worth highlighting the structural similarity of the [C_9_O_6_] core between the two [Ti_2_Pn†2] moieties found in (**2**) (Fig. 4 and 5), with the cyclic hexamers and octamers of carbon suboxide discussed in the Introduction (Fig. 1), all of which contain a 4-pyrone ring system.

**Fig. 6:**
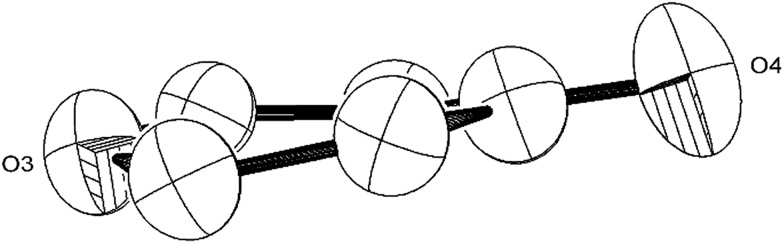
ORTEP diagram (50% probability ellipsoids) of the 6-member heterocycle found in the [C_9_O_6_] core of (**2**).

The Ti–Ti bonds in (**2**) (2.4648(14) and 2.4834(16) Å) are retained but have been slightly elongated in comparison to that in (**1**) (2.399(2) Å).^37^ As can be seen from Fig. 4, two titanium centres (Ti3 and Ti2) bind to two CO moieties in an η^2^ fashion. This bonding mode is best described as an haptoacyl with a considerable carbenoid contribution to the resonance structure. We base this on the observed metrics of the corresponding bond lengths and angles (Ti2–C1: 2.17(3) Å, Ti2–O: 2.13(3) Å, Ti3–C7: 2.316(17) Å Ti3–O: 2.133(19) Å, C1-01/C7–O5: 1.30(3)/1.28(3) Å; C7–Ti3–O: 33.1(7)°, C1–Ti2–O: 35.3(8)° C7–O–Ti3: 81.3(12)° Ti2–O–C1: 74.1(16)°) which compare well with those crystallographically determined for [(η^5^-C_5_H_5_)_2_Ti(η^2^-*CO*Me)Cl] (Ti–*C*: 2.07(2) Å, Ti–O: 2.194(14) Å, *C*–*O*: 1.18(2) Å; *C*–Ti–*O*: 32.0(4)°, Ti–*O*–*C*: 68.3(7)°, Ti–*C*–*O*: 79.7(6)°),^44^ as well as the ^13^C{^1^H}-NMR spectroscopic data discussed above. Furthermore, the bonding of these haptoacyl moieties to the pyrone heterocycle of the [C_9_O_6_] core (*i.e.* C2–C1 and C5–C7. 1.38(3) Å and 1.46(3) Å respectively) are in good agreement with the *CO*–CH_3_ groups found in [(η^5^-C_5_H_5_)_2_Ti(η^2^-*CO*Me)Cl] (*C*–C: 1.47(3) Å).^44^ A notable feature of the [C_9_O_6_] core in (**2**) is that the pyrone 6-membered ring is not planar (deviates from planarity by 0.055 Å, Fig. 6), and therefore lacks aromaticity. As a result, the C–C bond lengths of this 6 member ring are elongated in comparison to the ones found in 4-pyrone,^45^ although the C=O bond distances (*i.e.* C6–O4: 1.207(18) Å *vs.* 1.253(12) Å in 4-pyrone) are similar within esd's. Unfortunately, due to the mixed occupancy of the CCO moiety and O6 over the two sides of the [C_9_O_6_] core in (**2**) and the resulting crystallographic restraints used to model this disorder, we cannot talk with certainty about the bond lengths and angles of these two ligating moieties to this 6-member ring. Nevertheless, upon inspection of the corresponding bond lengths of these two atoms to the Ti centres, we can deduce that the bonding situation is far from straightforward. For instance, the Ti1–C′ bond resembles the ones found in Ti–NHC complexes, although closer to the high end of the spectrum (2.2–2.35 Å),^46^ and is in the same range as the ones discussed for the oxy-carbene moieties discussed above. A comparison with the corresponding lengths and angles found for free C_3_O_2_ 11 shows that the C′–C9 bond is elongated (1.2475(15) Å in free C_3_O_2_) while the C–O bond remains unchanged (1.442(13) Å in free C_3_O_2_). The same trend (*i.e.* C–C elongation) applies when compared with the corresponding bond lengths found in ketene (C=C: 1.314 Å, C=O: 1.162 Å).^47^ Similarly, the O6–Ti4 bond distance is closer to the ones found in the C=O–Ti dative interaction, *e.g.* in [*cis*-Ti(OEt_2_)_2_(η^2^-maltolato)_2_].^48^ (**2**) is diamagnetic and the elongation of the Ti–Ti bonds might suggest an increase of the formal oxidation state of each Ti centre by one (*i.e.* Ti(ii) in (**1**) to Ti(iii) in (**2**)). However, the oxy-carbene character of the C7–O5 and C1–O1 units as well as the non-aromatic 6 membered heterocycle of the [C_9_O_6_] core suggest that complicated resonance structures are in play, and assignment of formal oxidation state and Ti–Ti bond order in (**2**) is therefore problematic.

**Fig. 7:**
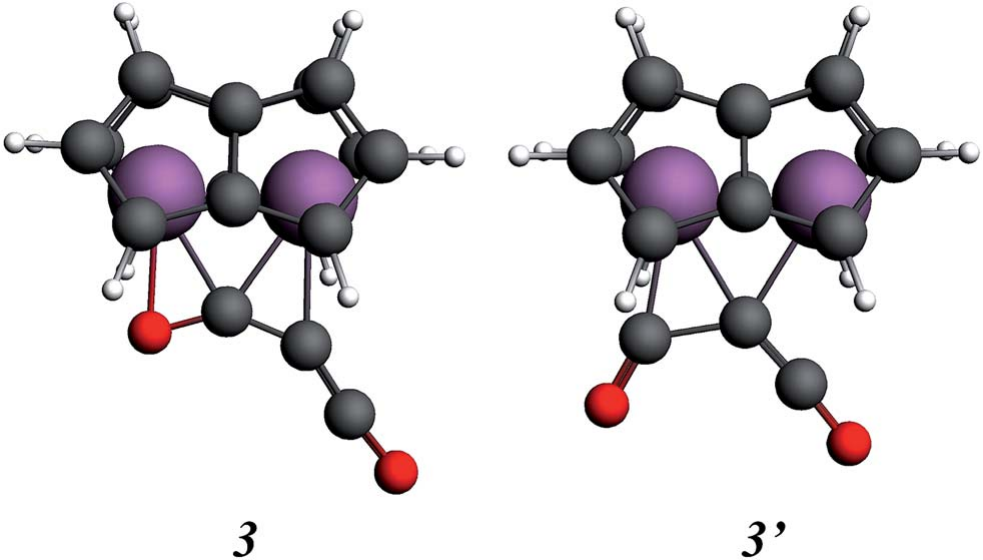
Calculated structures for the addition of C_3_O_2_ to model [Ti_2_(μ:η^5^,η^5^-Pn)_2_] (Pn = C_8_H_6_).

In order to gain a better understanding of the formation of (**2**), the reaction was probed computationally. Density functional calculations using the ADF program suite (BP86/TZP) were carried out on model systems in which the Si^i^Pr_3_ groups on the pentalene ligands were replaced by H atoms to increase computational efficiency. The computational analogues of experimental structures are denoted by italics; calculations on the analogue of the starting material **1**, Ti_2_(C_8_H_6_)_2_**1**, have been described previously.^37^,^40^ Geometry optimisation of the possible addition product of the first molecule of C_3_O_2_ to **1**, Ti_2_(C_8_H_6_)_2_(C_3_O_2_), led to two local minima, **3** and **3′** (Fig. 7).

Isomer **3** was the more stable being lower in energy by 0.59 eV, and resembled more closely the structure inferred from the disordered X-ray data (*vide infra*). The alternative structure, **3′**, could possibly be formed as a kinetic product. The structure of **3** clearly suggests that formation of **2**, with two C atoms and one O atom bound to the two Ti atoms, proceeds by the left hand side of the molecule depicted in Fig. 5 being the initial product rather than the right hand side where only one C atom and two O atoms are bound to the two Ti atoms.

**Fig. 8:**
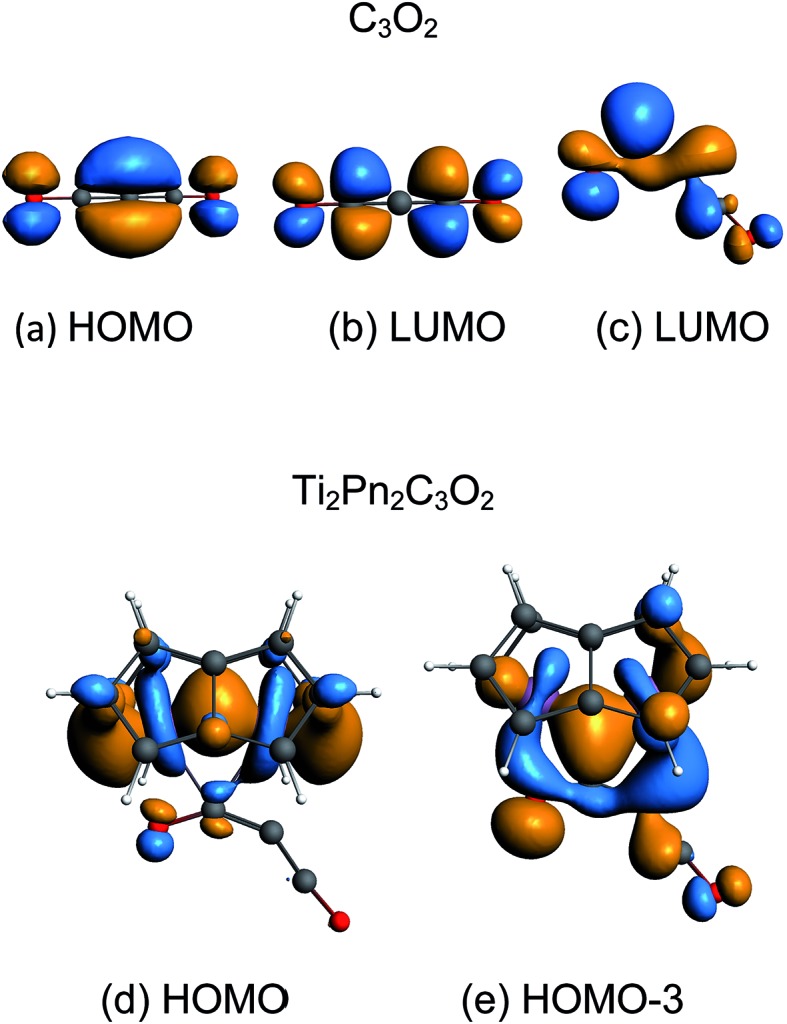
Key orbitals of linear and bent C_3_O_2_ and Ti_2_Pn_2_C_3_O_2_.

The coordination mode of C_3_O_2_ proposed here differs from some others calculated which indicate bonding primarily to the central carbon. In the cases of metal carbonyls^32^ and AuCl^33^ for example the metals function primarily as electron pair acceptors whereas Ti_2_Pn_2_ has very high energy electrons and acts as a electron pair donor through its Ti–Ti bond.^37^–^41^ The HOMO of C_3_O_2_ has electron density on the central C (Fig. 8a), hence this carbon is preferred for bonding by a Lewis acid, whereas the LUMO is localised on the outer carbons (Fig. 8b) leading to asymmetrical bonding by a Lewis base. Bending C_3_O_2_ to the geometry calculated for **3** concentrates the LUMO on an outer carbon (Fig. 8c).

The HOMO of **3** is a largely unperturbed Ti–Ti bonding orbital (Fig. 8d). The HOMO-1 and HOMO-2 correspond to TiPn bonding orbitals. The HOMO-3 (Fig. 8e) is responsible for C_3_O_2_ binding and is formed by donation from the other Ti–Ti bonding orbital into the LUMO of bent C_3_O_2_. A suitable analogy for the bonding situation in (**3**) can be found in that described for the adduct of (**1**) with CO_2_ [Ti_2_(μ:η^5^,η^5^-Pn^†^)_2_(μ-CO_2_)] (**6**) which has been studied computationally due to the instability of (**6**) in solution (one can conceptually replace the C_2_C_3_O_2_ moiety with O and *vice versa*).^40^ Indeed, a computational analysis of the orbitals involved in the coordination of C_3_O_2_ in (**3**) reveals a similar picture to the one found in (**6**). The Ti–O distance in **3** (2.19 Å) is shorter than that of **6** (2.27) indicating increased donation to O. This analogy between (**3**) and (**6**) is further reflected by the short Ti–Ti bond distances that are characteristic of both these computational models. It should be noted that the HOMO-3 retains Ti–Ti bonding character hence there is only a slight lengthening of Ti–Ti distance from **1** to **3** (2.37 Å to 2.41 Å). A CBC^49^ representation of **3** has an arrow going from the Ti=Ti double bond to C_3_O_2_ acting as a Z ligand, in the same way as CO_2_ behaves in [Ti_2_(μ:η^5^,η^5^-Pn^†^)_2_(μ-CO_2_)].^40^ The computed bond distances (ESI Table S2‡) of the coordinated C_3_O_2_ in this model are in good agreement with the ones determined crystallographically *vide infra*.

**Fig. 9:**
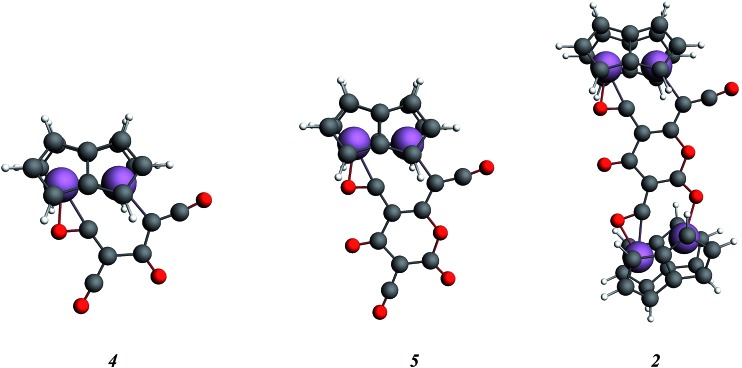
Optimised structures for the model key intermediates **4** and **5** leading to the formation of **2**.

In order to examine the energetics for the formation of **2**, and to investigate possible intermediates in the reaction, the geometries of Ti_2_(C_8_H_6_)_2_(C_3_O_2_)_2_, **4**, Ti_2_(C_8_H_6_)_2_(C_3_O_2_)_3_, **5**, and **2** were optimised (Fig. 9). Key bond lengths for all calculated species are given in the ESI (Table S2‡).

**Fig. 10:**
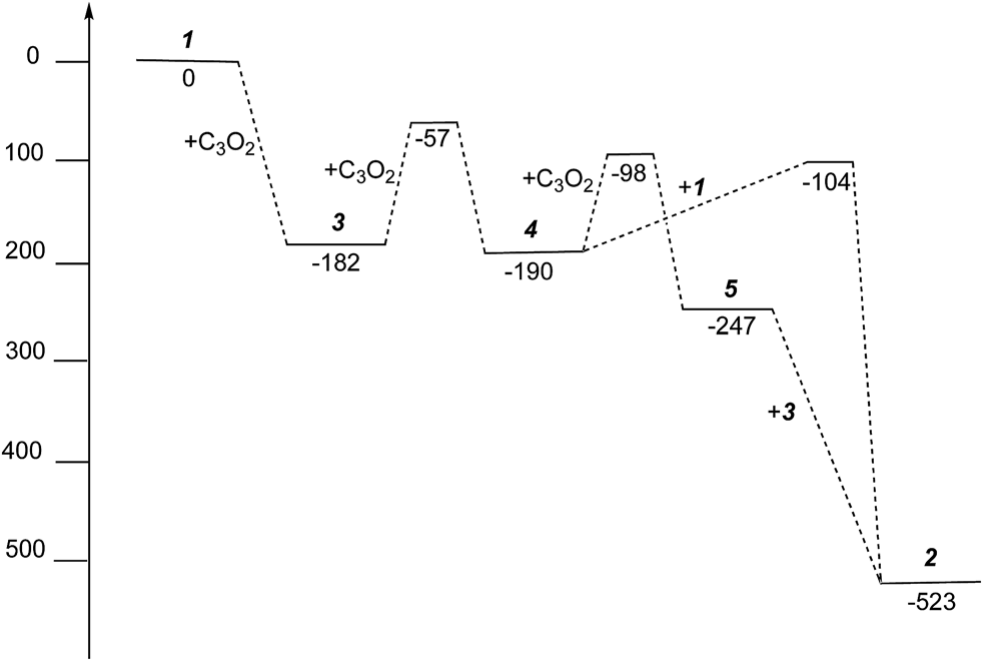
Gibbs free energies (kJ mol^–1^) for intermediates and activation energies (kJ mol^–1^) for proposed reaction pathways in the formation of **2** from **1** and C_3_O_2_.

The free energies for possible reaction pathways are shown in Fig. 10, and activation energies, where identified, are given in italics. The barriers to C_3_O_2_ and to **5** reacting with **1** appear to be purely entropic.

**Fig. 11:**
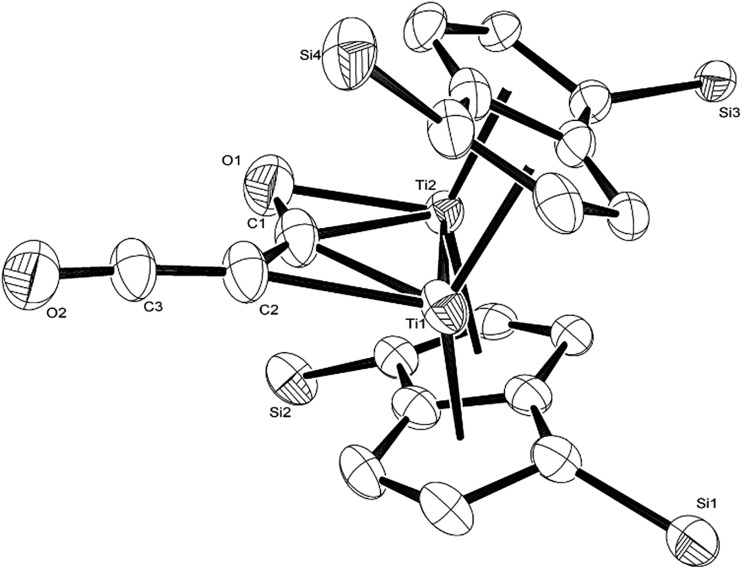
Molecular structure of (**3**) displaying 50% probability ellipsoids. Hydrogen atoms and ^i^Pr groups have been removed for clarity. Selected bond lengths (Å) and angles (°): Ti1–Ti2: 2.4293(14), Ti2–O1: 2.205(10), Ti2–C1: 2.148(5), Ti1–C1: 2.145(6), Ti1–C2: 2.304(10), C1–O1: 1.372(12), C1–C2: 1.291(13), C2–C3: 1.300(13), C3–O2: 1.175(9), O2–C3–C2: 172.0(10), C3–C2–C1: 132.5(10), C2–C1–O1: 137.0(7), C1–Ti1–Ti2: 55.58(15), C1–Ti2–T1: 55.49(16), O1–Ti2–T1: 92.2(3), C1–Ti2–O1: 36.7(3), Ti2–C1–Ti1: 68.92(17), C1–C2–Ti1: 66.5(5), Ti1–C2–C3: 161.0(9), C1–O1–Ti2: 69.4(4).

The calculated mechanism highlights two key steps in the formation of (**2**): (a) the formation of the intermediate [Ti_2_(μ:η^5^,η^5^-Pn^†^)_2_(μ:η^2^,η^1^-*OCC*′CO)] (**3**) (*i.e.* the adduct between (**1**) and C_3_O_2_ that would arise from the addition of 1 eq. of the latter to the former) and (b) the termination of the consecutive two additions of C_3_O_2_ to (**3**) by the capping of (**5**) by (**1**) or alternatively (c) the reaction of (**4**) with (**3**). The barriers for the two pathways, (b) and (c), are too similar to distinguish between them energetically. In all possible pathways leading to (**2**), the formation of adduct (**3**) is the common denominator. The high activation energy calculated for the reaction of (**3**) with a further molecule of C_3_O_2_ to form (**4**) indicates that (**3**) should be isolable at low temperature. Indeed, repeating the reaction in the same manner as for the synthesis of (**2**), but removing volatiles at *ca.* 0 °C, resulted in the formation of no carbon sub-oxide polymer and ^1^H-NMR analysis showed the formation of an extra species along with (**2**), exhibiting two inequivalent pentalene ligand scaffolds (*i.e.* 8 doublets in the aromatic region). Encouraged by this observation, the reaction between (**1**) and C_3_O_2_ was repeated under higher dilution conditions to prevent the last step of the formation of (**2**) and the reaction mixture was kept below –10 °C throughout. Upon removing volatiles at low temperature (*ca.* –25 °C), and lyophilising the residue with benzene (below –10 °C), this new species was isolated in almost quantitative yields and with spectroscopic purity of >98%. More conclusive evidence that (**3**) is indeed that predicted by calculations was provided by ^13^C{^1^H}-NMR spectroscopy. The most salient features of this spectrum are three resonances located at 159.8, 260.4 and 7.03 ppm which all correspond to quaternary carbons and which we assign to coordinated C_3_O_2_ (for free C_3_O_2_*δ*(CDCl_3_, –40 °C): 129.74 (O*C*C*C*O) and –14.62 (OC*C*CO)^50^). The two downfield resonances are assigned to the terminal CO's with the one at 159.8 ppm assigned to an un-coordinated CO moiety and the one at 260.4 ppm to a coordinated one. The former is in good agreement with previously reported values reported by Hillhouse *et al.* and Pandolfo *et al.* using ^13^C-CP/MAS NMR spectroscopy, while the latter is significantly shifted downfield in comparison with these two literature examples (187.8 and 179.7 ppm for [M(η^2^(*C*,*C*′)-C_3_O_2_)(PPh_3_)_2_] with M = Ni, Pt respectively).^29^,^51^ Similarly the central carbon in the coordinated C_3_O_2_ in (**3**) is found at much lower field (7.03 ppm) compared with the ones assigned to the terminal carbons (see above) and follows the trend observed in previous studies (–12.3 and –16.2 ppm for [M(η^2^(*C*,*C*′)-C_3_O_2_)(PPh_3_)_2_] with M = Ni, Pt respectively); it has to be noted though that is shifted downfield compared to these reported values. These discrepancies are expected as the documented examples concern electron rich monometallic metal fragments of d^10^ transition metals, unlike the present case where a *syn*-bimetallic Ti–Ti core is involved. The coordination of C_3_O_2_ was further corroborated by IR spectroscopy (thin film) that showed characteristic bands for CCO (2060 cm^–1^) and CO functionalities (1588, 1575 and 1510 cm^–1^) (for free C_3_O_2_ 2280 cm^–1^) which are in good agreement with values reported for the complexes [M(η^2^(*C*,*C*′)-C_3_O_2_)(PPh_3_)_2_] (M = Ni, Pt).^28^,^51^ Unfortunately, mass spectrometry was not informative and microanalysis was hampered by the thermal instability of (**3**) even in the solid state. Nevertheless, based on the spectroscopic data discussed above, (**3**) was assigned as the adduct of C_3_O_2_ with (**1**), *i.e.* the first intermediate towards the formation of (**2**). This was unequivocally established by a single crystal XRD study (Fig. 11).

As can be seen from Fig. 9, C_3_O_2_ coordinates *via* one of the terminal CO's in an η^2^ fashion to one of the Ti centers (Ti2) and η^1^*via* that same carbon to the other one (Ti1). The latter also coordinates to the central carbon (C2) of the C_3_O_2_ ligand. The molecular structure of (**3**) represents the first example of a crystallographically authenticated example of C_3_O_2_ coordination and confirms the coordination modes of C_3_O_2_ predicted by Pandolfo and Hillhouse based on spectroscopic evidence.^28^,^51^ However the coordination mode calculated for M(PH_3_)_2_C_3_O_2_ (ref. 44) is closer to that found for **3′** where an O is not coordinated. Presumably the bimetallic nature of Ti_2_Pn_2_ allows more extensive donation to the unsaturated substrate. The Ti–Ti bond in (**3**) (2.4293(14) Å) is similar to the one found in parent (**1**) (2.399(2) Å) within esd's; a similar invariance in the Ti–Ti bond length has been observed in the adducts of (**1**) with CO ([Ti_2_(μ:η^5^,η^5^-Pn^†^)_2_(μ:η^2^,η^1^-CO)] *d*_Ti–Ti_ = 2.4047(5) Å; [Ti_2_(μ:η^5^,η^5^-Pn^†^)_2_(CO)_2_] *d*_Ti–Ti_ = 2.4250(10) Å).^40^ The ligation of C_3_O_2_ has a profound effect on its bond angles, with the most prominent changes being the significant deviation of the O1–C1–C2 and C1–C2–C3 angles from linearity (179.93(11)° and 178.32(12)° respectively in free C_3_O_2_ 11) to 137.0(7)° and 132.5(10)° respectively. The C2–C3–O2 also deviates from linearity (172.0(10)° *vs.* 179.57(12)° in free C_3_O_2_ 11) but to a much lesser extent. On the other hand, the bond distances in the ligated C_3_O_2_ are similar within esd's to the ones found in free C_3_O_2_ (C1–O1: 1.372(12)/1.1479(12) Å; C1–C2: 1.291(13)/1.2564(15) Å; C2–C3: 1.300(13)/1.2475(15) Å; C3–O2: 1.175(9)/1.1442(13) Å) with the exception of the C1–O1 bond distance which is elongated (1.372(12) Å in (**3**) *vs.* 1.1479(12) Å in free C_3_O_2_ 11). In comparison to the CCO moiety found in (**2**) (Fig. 4), the corresponding C–C bond (*i.e.* C2–C3) is shorter (1.300(15) Å *vs.* 1.40(3) Å in (**2**)) while the C–O bond lengths are the same within esd's. In the case of the corresponding angles, the CCO angle in both (**2**) and (**3**) are identical (172.4(16) Å and 172.0(10) Å respectively) (Fig. 11).

In conclusion, we report the first example of the trimerisation of C_3_O_2_ promoted by a well-defined molecular complex leading to the formation of (**2**). The core structure between the two [Ti_2_Pn†2] moieties is reminiscent of biologically relevant compounds responsible for the regulation of ion concentrations in cells. This transformation was studied computationally revealing that the first step is the formation of (**3**), which was confirmed experimentally by its isolation and structural characterization.

## Conflicts of interest

There are no conflicts of interest to declare.

## Supplementary Material

Supplementary information

Crystal structure data
