# Supplementary material for: Trimerisation of carbon suboxide at a di-titanium centre to form a pyrone ring system
Source: Chem Sci. 2018 May 8;9(22):5008–14. doi: 10.1039/c8sc01127c (PMC5994745; doi:10.1039/c8sc01127c)
Supplement: Supplementary file 1 [file SC-009-C8SC01127C-s001.pdf]

Electronic Supplementary Material (ESI) for Chemical Science.
This journal is © The Royal Society of Chemistry 2018

SUPPLEMENTARY INFORMATION

Trimerisation of Carbon Suboxide at a Di-titanium Centre to form a Pyrone Ring System

Nikolaos Tsoureas, Jennifer C. Green, F. Geoffrey N. Cloke, Horst Puschmann, S. Mark Roe and Graham Tizzard

EXPERIMENTAL

General Methods

All manipulations were carried out in a MBraun glovebox under N_2 or Ar (O_2 and H_2O <1 ppm) or by using standard Schlenk techniques under Ar (BOC pure-shield) passed through a column containing BASF R3-11(G) catalyst and activated molecular sieves (4 Å). All glassware was dried at 160 °C overnight prior to use. Filter cannulas were prepared using Whatman DG/F 25 mm glass microfiber filters that were pre-dried at 160 °C overnight. Hydrocarbons were dried over molten K (toluene) or $NaK_{2.8}$ (n-pentane, n-hexane, n-heptane) distilled under a N_2 atmosphere and kept in a Young's ampules over a potassium mirror under Ar. $(SiMe_3)_2O$ was degassed and pre-dried over activated molecular sieves (4 Å), followed by stirring over $NaK_{2.8}$ for 2 days before being vacuum distilled and kept over activated molecular sieves. Deuterated toluene and benzene, were degassed by three freeze−thaw cycles, dried by refluxing over molten K for 3 days, vacuum distilled, and kept in Young's ampoules in the glovebox under N_2. $Ti_2(\mu{:}\eta^5,\eta^5\text{-Pn}^\dagger)_2$ ($\text{Pn}^\dagger = C_8H_4(1,4\text{-Si}^i\text{Pr}_3)_2$) (**1**) (abbreviated as [Ti_2]) was prepared according to published procedures[1] and stored in a glovebox freezer (-35 ºC) under N_2. Carbon suboxide was prepared by a modification of the literature method (a very detailed description, complete with images of the apparatus, is given below).[2] ^{1}H-NMR, ^{13}C{^{1}H}-NMR, ^{29}Si{^{1}H}-NMR and correlation experiments were obtained on a Varian VNMR S400 or Varian S500 spectrometers operating at 400 and 500 MHz (^{1}H) respectively at 30 °C unless otherwise stated, using Young's NMR tubes. The spectra were referenced internally to the residual protic solvent (^{1}H) or the signals of the solvent (^{13}C). ^{29}Si{^{1}H} NMR spectra were referenced externally relative to $SiMe_4$. EI-MS mass spectra were recorded on a VG-Autospec Fisons instrument at the University of Sussex unless otherwise stated. IR spectra were recorded on a Perkin Elmer 100 FT-IR instrument as thin films in the range 4000-600 cm^{-1}. Elemental analyses were performed by Microanalytisches Labor Pascher GmbH.

Synthesis of [Ti_2](C_3O_2)$_3$[Ti_2] (2)

In a typical procedure a 300 mL ampoule equipped with a greaseless tap was charged with 100 mg of (**1**) (0.108 mmol) in a glovebox and dissolved in 2-3 mL of toluene to produce a crimson red solution. This was cooled at -78 ˚C, connected to a high vacuum line and evacuated. The ampoule was then exposed to the contents of a second ampoule containing pure C_3O_2 also held at -78 ˚C (at which temperature the vapor pressure of C_3O_2 is 6 mmHg, hence resulting in the addition of *ca.* 0.12 mmol of C_3O_2 to the ampoule containing the solution of (**1**)) for a few minutes. The ampoule containing the reaction mixture was then isolated and any C_3O_2 in the gas phase condensed into the solution of (**1**) by briefly cooling the very bottom of the ampoule in liquid nitrogen. The reaction mixture immediately adopted a deep brown-green coloration, and the ampoule was then placed in a -35 ˚C slush bath and slowly allowed to warm to 17 ˚C with vigorous stirring (typically 2-3 hrs). When the temperature

reached 17 ˚C, the formation of brown-red precipitates was observed, and the volatiles were removed under vacuum and the solids dried. They were then extracted with n-pentane (10 mL + 2 x 5 mL), filtered and volatiles removed to give the title compound as a brown-green film that can be further isolated as a brown-green powder after lyophilization with C_6H_6 (*ca* 1-2 mL) and which is pure by NMR spectroscopy. An analytically pure sample was obtained by washing this solid with $(SiMe_3)_2O$ in which (**2**) is sparingly soluble, filtering and drying. Yield: 50-70 mg (50-66%). *NOTE:* The yields vary based on the final temperature of the reaction mixture: we have observed that allowing the temperature of the reaction mixture to increase above 17 ˚C results in decreased yields. Similarly, when the work-up of the reaction mixture starts between 0-15 ˚C, mixtures of (**2**) and (**3**) are isolated. Crystals suitable for single crystal X-ray diffraction can be obtained by partial evaporation of an n-pentane solution (*ca* 0.5-1.0 mL) under a stream of Ar, followed by refrigeration (-40 ˚C). Elem. Anal. (calcd., found) for $C_{113}H_{184}O_6Si_8Ti_4.(SiMe_3)_2O$: C (64.46, 64.12), H (9.18, 8.73); IR (thin film, cm^{-1}): 2061.9 (s, ν_{CCO}), 1658.3, 1591.2, 1532.4 (ν_{CO}); ^{1}H-NMR δ (400MHz, C_6D_6): 8.19 (d, 1H, $^3J_{HH}$ = 3.50 Hz, Pn*H*), 8.17 (d, 1H, $^3J_{HH}$ = 2.72 Hz, Pn*H*), 7.90 (d, 1H, $^3J_{HH}$ = 2.72 Hz, Pn*H*), 7.62 (d, 1H, $^3J_{HH}$ = 3.11 Hz, Pn*H*), 7.46 (d, 1H, $^3J_{HH}$ = 3.89 Hz, Pn*H*), 7.40 (d, 1H, $^3J_{HH}$ = 3.50 Hz, Pn*H*), 6.48 (d, 1H, $^3J_{HH}$ = 3.50 Hz, Pn*H*), 6.46 (d, 1H, $^3J_{HH}$ = 3.11 Hz, Pn*H*), 6.44 (d, 1H, $^3J_{HH}$ = 2.72 Hz, Pn*H*), 6.40 (d, 1H, $^3J_{HH}$ = 3.11 Hz, Pn*H*), 6.38 (d, 1H, $^3J_{HH}$ = 3.50 Hz, Pn*H*), 6.32 (d, 1H, $^3J_{HH}$ = 3.50 Hz, Pn*H*), 6.30 (d, 1H, $^3J_{HH}$ = 3.50 Hz, Pn*H*), 6.23 (d, 1H, $^3J_{HH}$ = 2.73 Hz, Pn*H*), 5.82 (d, 1H, $^3J_{HH}$ = 3.50 Hz, Pn*H*), 5.69 (d, 1H, $^3J_{HH}$ = 3.50 Hz, Pn*H*), 1,70 (m, 12H, $Si(CH(CH_3)_2)_3$), 1.58 (m, 12H, $Si(CH(CH_3)_2)_3$), 1.38-1.08 (m, 134H, $Si(CH(CH_3)_2)_3$), 1.02 (d, 5H, J_{HH} = 1.56 Hz, $Si(CH(CH_3)_2)_3$), 1.00 (d, 5H, J_{HH} = 1.56 Hz, $Si(CH(CH_3)_2)_3$); $^{13}C\{^1H\}$-NMR δ (125.7 MHz, C_6D_6): 388.8, 388.6, 388.4, 275.1, 261.6, 178.8, 174.3, 167.6, 155.9, 140.6, 140, 137.3, 137.2, 132.4, 131.9, 131.4, 130.2, 129.3, 126.8, 126.1, 126.00, 125.7, 123.2, 120.1, 118.1, 117.7, 117.4, 112.7, 110.9, 110.7, 110.8, 109.7, 109.5, 109.6, 109.1, 105.1 104.9, 99.5, 98.5, 98.3, 96.8, 40.05, 20.05 (broad, Si^iPr_3), 14.28 (broad, Si^iPr_3), 12.88 and 12.67 (broad, Si^iPr_3); $^{29}Si\{^1H\}$-NMR δ (79.5 MHz, C_6D_6): 1.60, 1.79, 2.31, 2.44, 3.37, 3.54, 3.81 (1:1:1:1:2:1:1 relative integration respectively); MS: No molecular ion could be observed.

Synthesis of $[Ti_2](C_3O_2)$ (3)

Similarly to the synthesis of (**2**), the ampoule was charged with 96 mg of (**1**) (0.104 mmol) which was dissolved in *ca* 20 mL of n-pentane and *ca*. 0.1 mmol C_3O_2 added. The reaction mixture instantly adopted a brown-green coloration, and was was placed in a -78 ˚C bath and allowed to slowly warm to -35 ˚C to -25 ˚C, at which point volatiles were slowly removed under vacuum. This produced (**3**) as a brown film (in almost quantitative yield and with >98% spectroscopic purity (*ca* 2% of (**2**)) that was further dried for *ca* 10 minutes at RT and which was then lyophilised between -10 ˚C and 0 ˚C with C_6H_6 to isolate it as an extremely air-sensitive brown solid. Compound (**3**) is not very stable as a solid

or in solution for long periods of time at RT but is stable as a solid if kept ≤ 5 ˚C. Crystals suitable for single crystal X-ray diffraction were obtained by the slow evaporation of n-pentane at -35 ˚C. Elem Anal.: Due to the thermal instability of (**3**), no combustion analysis could be obtained; IR (thin film, cm^{-1}): 2060.2 (s, ν_{CCO}), 1588.8, 1575.38, 1510 (w, ν_{CO}); ^{1}H-NMR δ (400MHz, C_6D_6): 6.98 (d, 1H, $^{3}J_{HH}$ = 3.02 Hz, Pn*H*), 6.91 (d, 1H, $^{3}J_{HH}$ = 3.54 Hz, Pn*H*), 6.88 (d, 1H, $^{3}J_{HH}$ = 3.03 Hz, Pn*H*), 6.51 (d, 1H, $^{3}J_{HH}$ = 3.02 Hz, Pn*H*), 6.49 (d, 1H, $^{3}J_{HH}$ = 3.01 Hz, Pn*H*), 6.44 (d, 1H, $^{3}J_{HH}$ = 3.62 Hz, Pn*H*), 6.16 (d, 1H, $^{3}J_{HH}$ = 3.51 Hz, Pn*H*), 6.01 (d, 1H, $^{3}J_{HH}$ = 3.51 Hz, Pn*H*), 1.6-0.95 (m, 84H, Si$^{i}Pr_3$); ^{13}C{^{1}H}-NMR δ (100.6MHz, C_6D_6): 260.4 and 159.8 (C_3O_2), 134.3, 133.5, 131.7, 131.3, 129.4, 128.8, 126.1, 124.5, 123.1, 119.7, 107.3, 104.9, 104.4, 103.4, 96.32, 93.46 (Pn), 34.49 (n-pentane, CH_2), 22.76(n-pentane CH_3), 19.94 (broad), 19.77, 19.49, 19.17, 14.31 (n-pentane, CH_2) 14.19 (broad) 13.34, 13.23, 12.52, 12.25 (Si$^{i}Pr_3$), 7.03 (s, OC*C*CO); ^{29}Si{^{1}H}-NMR δ (79.5MHz, C_6D_6): 3.45 and 2.30 (*Si*$^{i}Pr_3$) (1:3 relevant integration respectively); MS: No molecular ion could be observed.

Single Crystal X-ray Diffraction. The Crystallographic Information Files (1817210-1817211) for (**2**) and (**3**) have been deposited in the Cambridge Crystallography Database.

Detailed Synthesis of C_3O_2: *Carbon suboxide has a strong pungent smell and is lachromatory.*

This was prepared by modification of the preparation described in the *Handbook of Preparative Inorganic Chemistry by Brauer*, as follows: 20 g of malonic acid, 50 g of calcined sand and approximately 250 g of P_2O_5 were mixed thoroughly and placed in a one liter flask equipped with a Young's tap take-off leading to a greaseless ball joint. Glass wool was placed into the adaptor connecting the flask to the takeoff. The flask and its contents, were placed under dynamic vacuum overnight (Fig. S1).

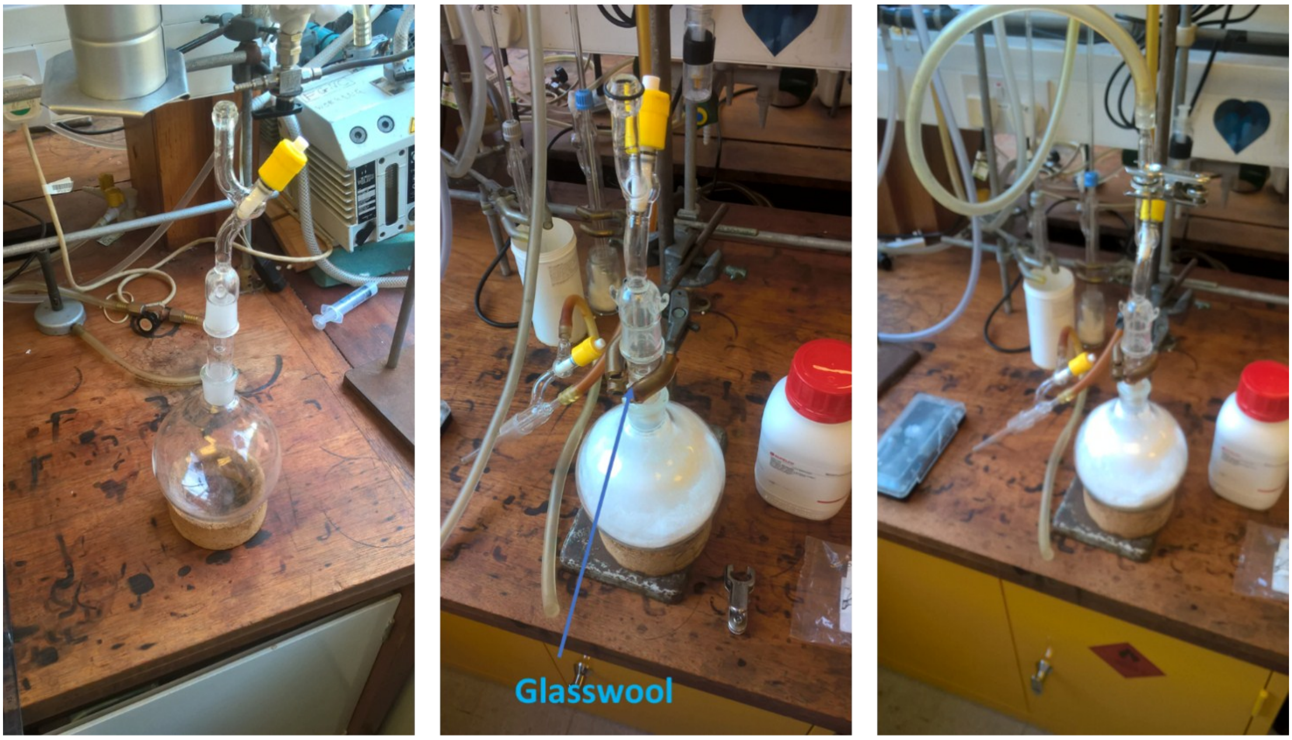

Figure S1

It was then connected to a vacuum manifold (see pictures below, Figure S2) and the whole system evacuated.

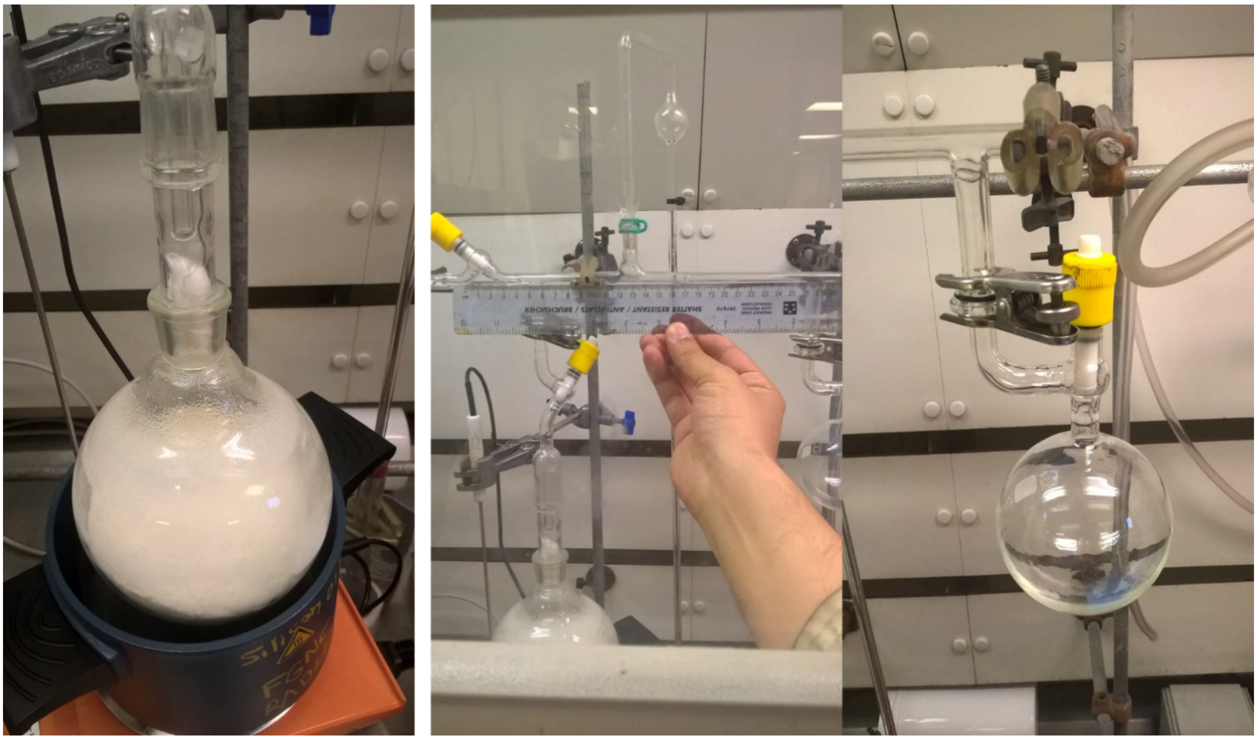

Figure S2: Setup before heating the solid mixture of malonic acid/sand/P_2O_5. Evacuate the 250 mL collection flask (right) and the vacuum bridge before opening the Young's tap on the take-off of the solid reaction mixture (left under dynamic vacuum overnight to (a) prevent P_2O_5 from reacting with moisture and (b) reduce the time of evacuation at this point).

After evacuation, the collection flask was placed in liquid N_2 and the solid reaction mixture was heated at 120-140 °C using a thermostatted silicone oil bath for 3-4 hr (start at 120 °C for 1 hour, 130 °C for 2 hours and 140 °C for 1 hour). The evolved gases (C_3O_2, CO_2 and CH_3COOH) were condensed into the liquid N_2 (-196 °C) cooled collection flask **under static vacuum** (Figure S3).

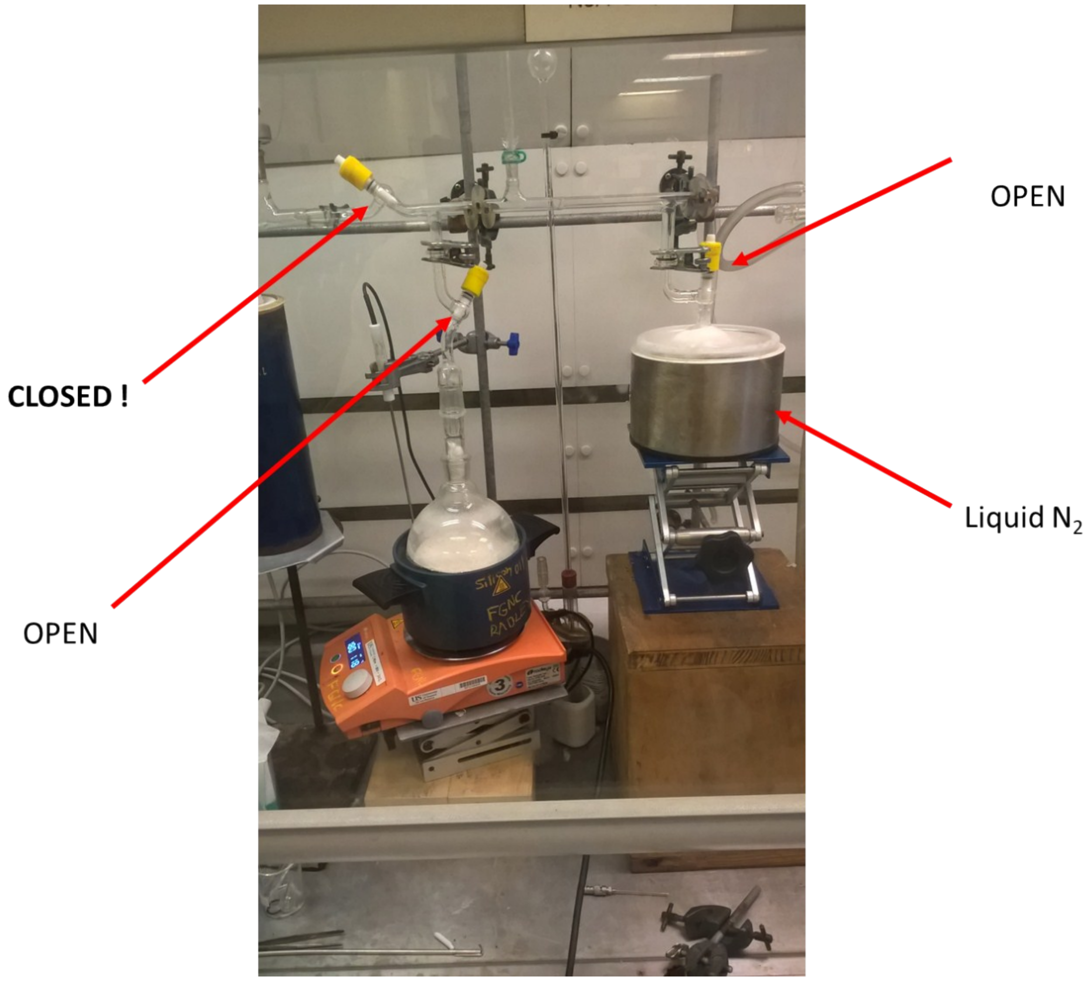

Figure S3

After the end of this period the collection flask on the right (Figure S3) was isolated and the reaction flask on the left containing the spent P_4O_{10} and sand was detached from the vacuum manifold. A 500 ml RBF equipped with a Young's adaptor attached to a CaO (**lumps**) scrubber column (which had been in an 160 °C oven overnight, flame-dried under vacuum for *ca* 20 minutes and kept under Ar), was evacuated and cooled at liquid N_2. The contents of the collection flask from the previous step, (on the right Figures 3 and 4) were cooled at -35 - -40 °C using a liquid N_2/MeCN slush bath and transferred through the CaO scrubber (to remove acetic acid and some of the CO_2) into the 500 ml flask **under static vacuum** (Figure S4).

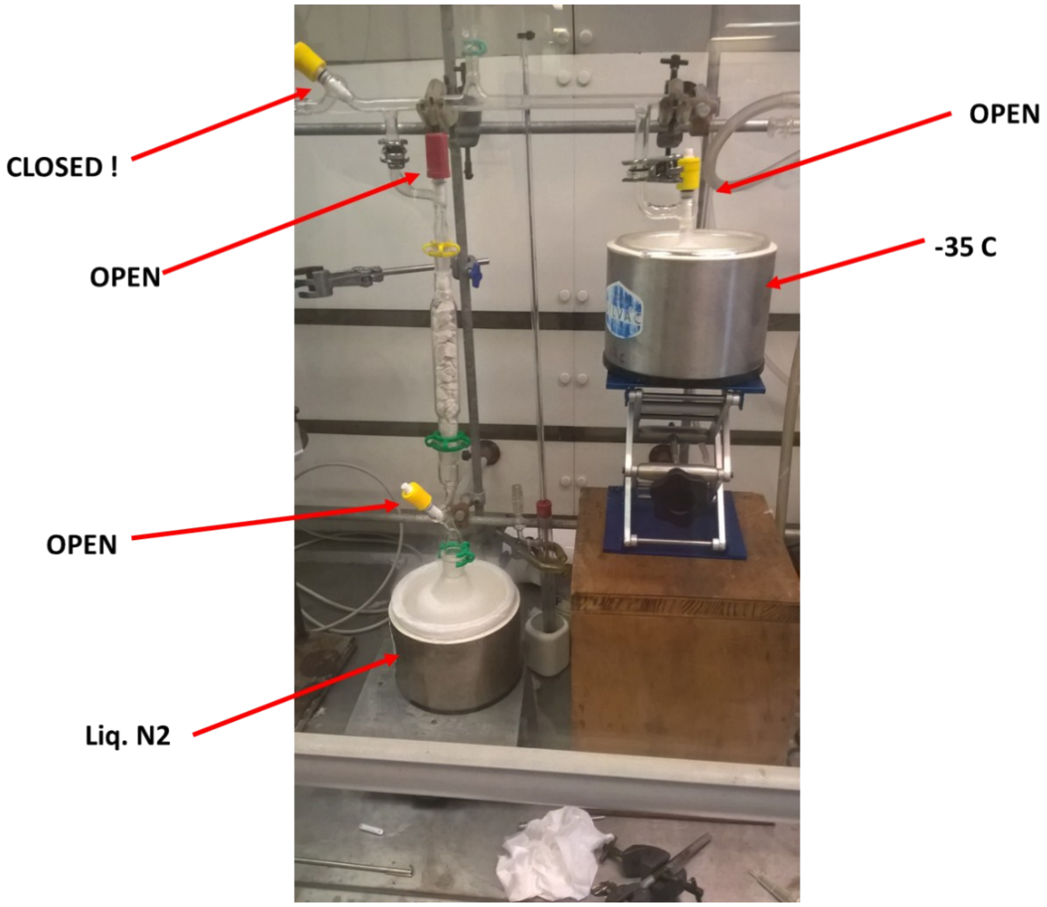

Figure S4

After the transfer was complete the scrubber was removed and the flask containing CO_2 and C_3O_2 was reattached to the vacuum manifold *via* an adaptor (Figure 5, picture on the left). Before the removal of CO_2 commenced, the final collection ampoule where C_3O_2 is stored was degassed (ROTAFLO ampoule on the right in Figure S5).

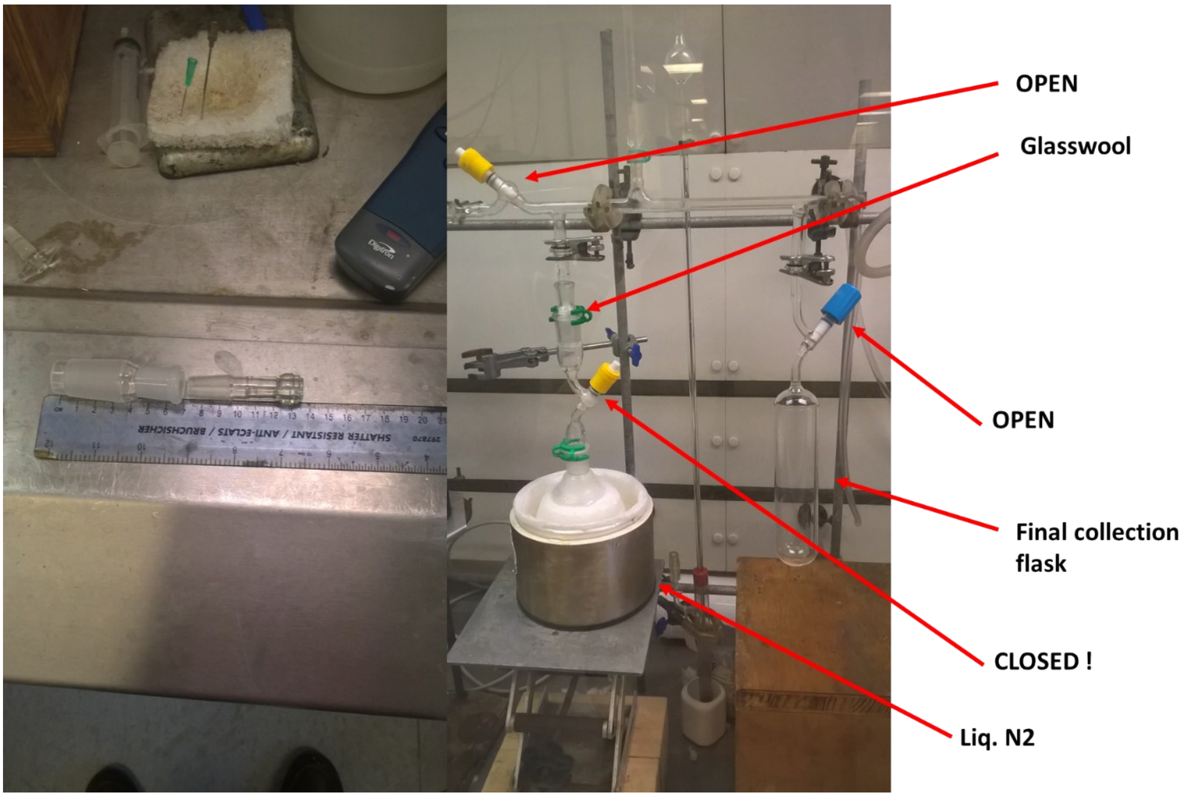

Figure S5: Set-up before the removal of CO_2 from C_3O_2.

The contents of the flask on the left were cooled at -105 - -120°C using a liquid N_2/n-pentane slash bath. With the flask cooled to *ca* -110 °C the CO_2 was removed under dynamic vacuum for *ca* 15-20 minutes (pirani gauge reads 8-7 x10^{-3} mbar) (Figure S6).

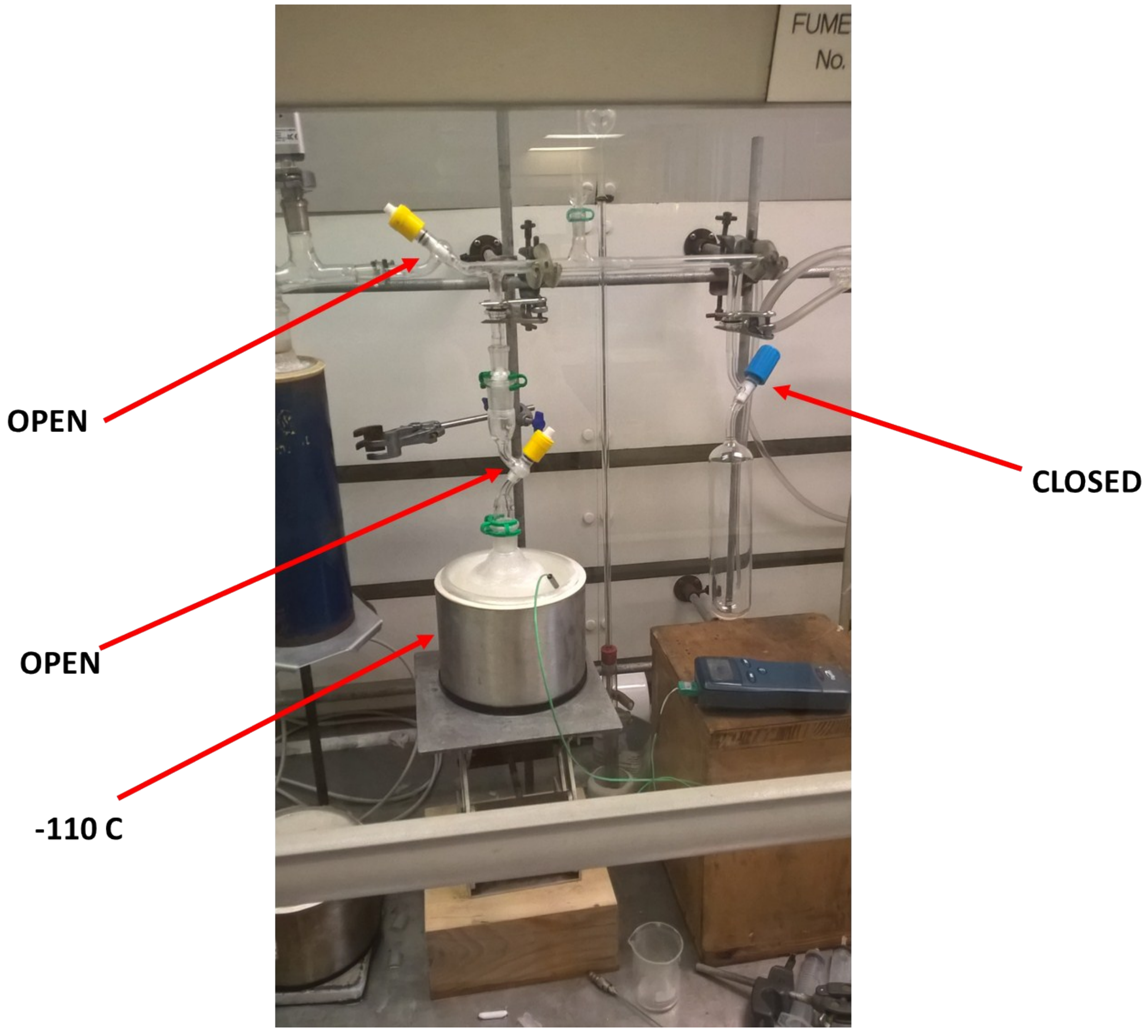

Figure S6: Removal of CO_2.

The flask on the left was then placed in a -78 °C slash bath (dry-ice/acetone) and the pressure measured with a manometer (at -78 °C the vapor pressure of C_3O_2 is 5 torr.) under static vacuum.

If the vapour pressure was more than 5 torr the previous step was repeated (*ie* cool at -120°C and pump off remaining CO_2). After removal of CO_2 was achieved, the final receiving ampoule (ROTAFLO ampoule on the right-hand side – Figure 5 and 6) was placed in liquid N_2, and the C_3O_2 transferred **under static vacuum** (Figure S8).

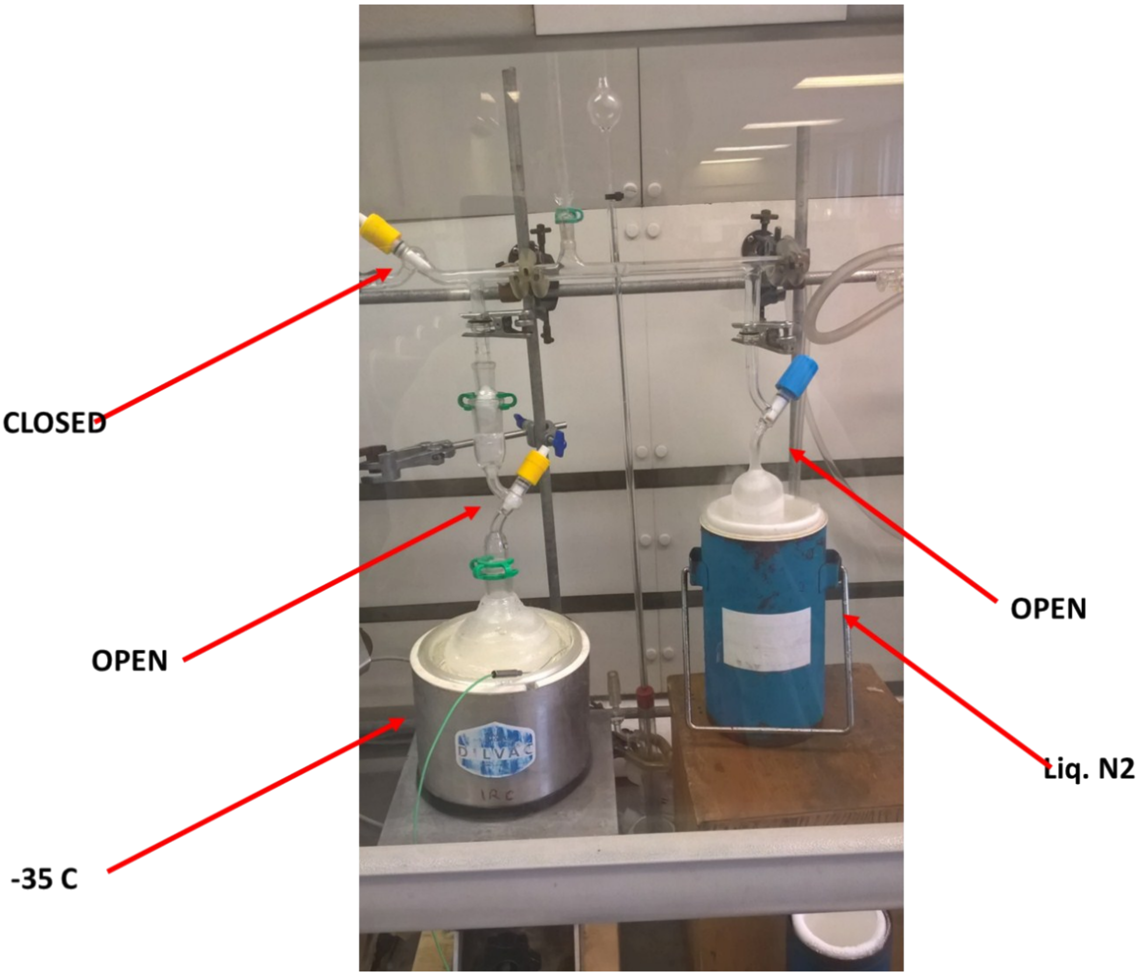

Figure S8: Final transfer of C_3O_2.

C_3O_2 is then kept in a well-insulated Dewar at -78°C using solid dry-ice. Typically, this preparation produces 1 to 2 mL of liquid C_3O_2 (-78 °C).

Pictures of some glassware and experimental setup:

We used Kalrez O-rings (per-fluorinated elastomer that is very chemically resistant). If greased taps are used, hydrocarbon lubricant should be avoided as C_3O_2 reacts with it but not with silicone grease.

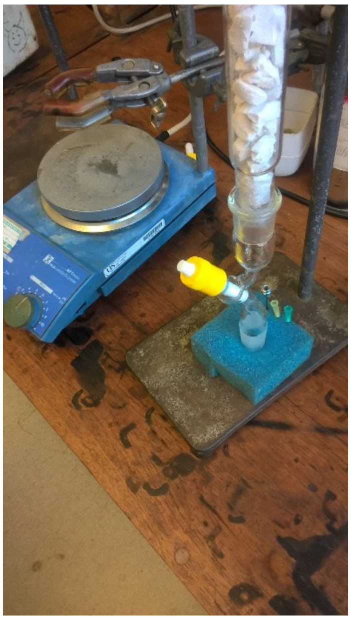

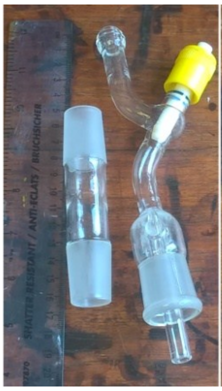

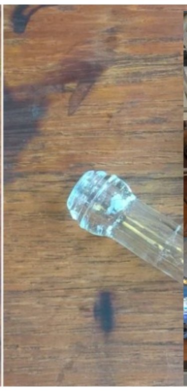

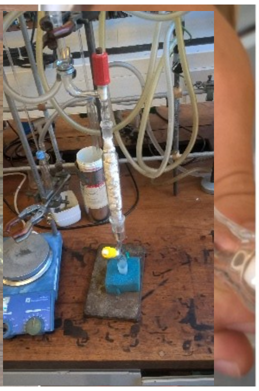

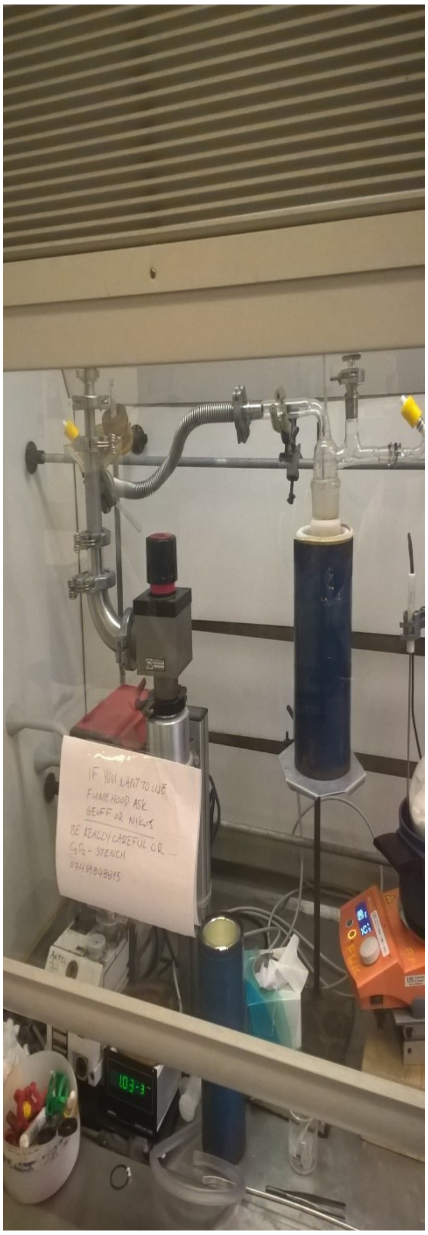
IF YOU WANT TO USE
FUME HOOD ASK
GEOFF OR NIKOS
BE REALLY CAREFUL OR ...

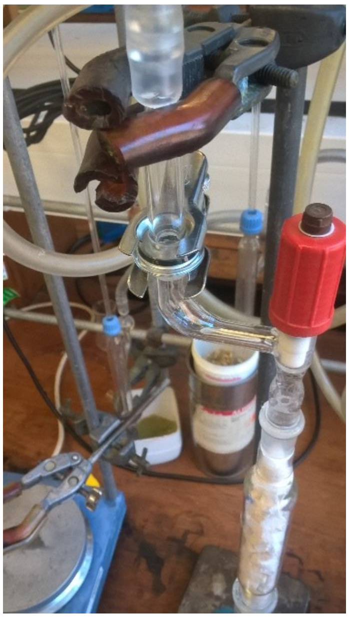

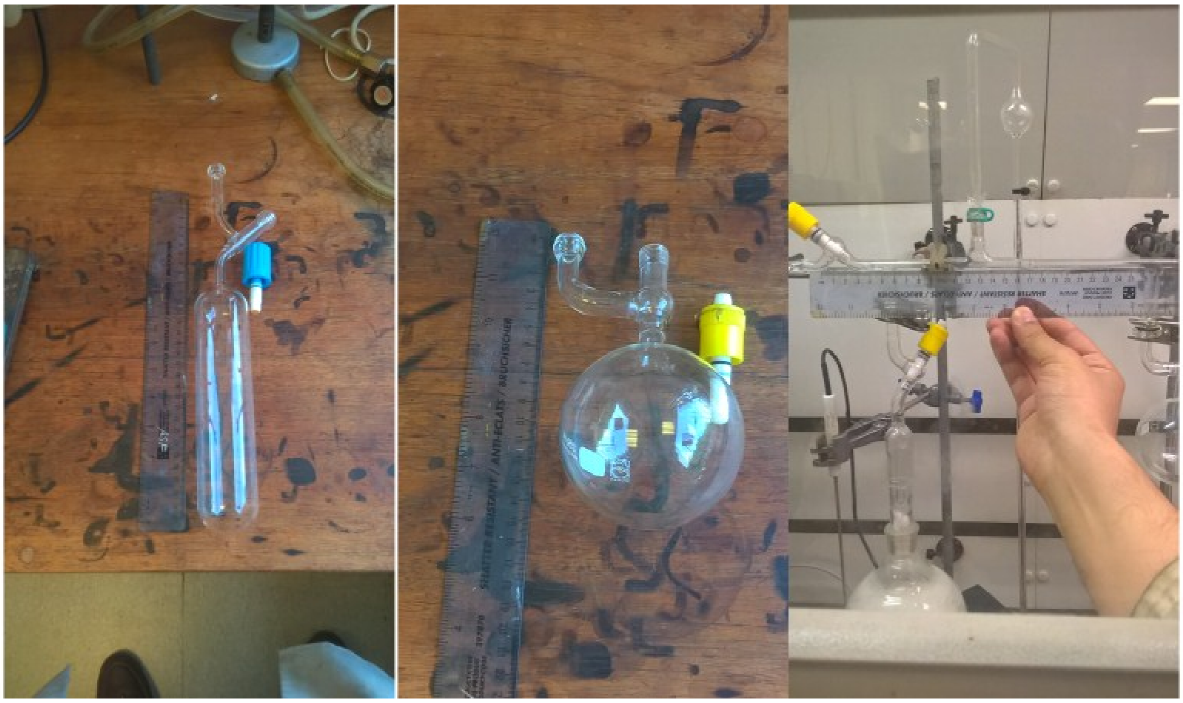

X-ray Crystallography:

Crystals were mounted on MitiGen cryoloops from dried Fomblin oil kept over 4Å molecular sieves in an Ar MBraun glovebox. All data were collected at 100 K. Data for (**2**) were collected on a Rigaku Micromax 007HF rotating anode diffractometer (Cu *Kα*), equipped with a ¼ chi goniometer, Varimax HF confocal mirrors and a Rigaku Saturn 724 CCD area detector in ω scanning mode to fill the Ewald sphere. In the case of (**3**), data were collected at the EPSRC National Crystallography Service at the University of Southampton (U.K.)[3] using a Rigaku FRE+ rotating anode source (Mo *Kα*) equipped with an AFC12 four circle goniometer, Varimax HF confocal mirrors and a Rigaku Saturn 724+ CCD area detector operating in ω scanning mode to fill the Ewald sphere. Data collection, indexing, data reduction and integration were handled by the CrysAlis Pro software suite in both cases. Structure solution and refinement were handled using the WinGX or Olex2 programs and all software packages within.[4,5] Hydrogen atoms were added using a riding model. All other atoms were refined using anisotropic thermal parameters. In the case of (**2**) repeated attempts to acquire better quality data from different crystals or by recrystallising the compound from a variety of solvents failed. The trimerised suboxide moiety is disordered over two positions resulting in the super-position of one of the CO moieties with the CCO one. These two residues were modelled using 50:50 occupancy of the two sites using the RESI command. Attempts to model the disorder freely (80:20) were also undertaken with the no substantial improvement to the model (in the latter case heavy use of SADI restraints was

necessary to provide a stable refinement). In the case of (**3**) the coordinated C_3O_2 is disordered over two positions resulting in mixed occupancy of carbon and oxygen atoms in certain positions (70:30- see respective crystallographic information file for more information). This disorder was modelled by using the PART command in SHELX and atoms were refined using SADI constraints where necessary. Crystal structure, data collection and refinement details of compounds (**2**) and (**3**) are provided in the following table (CCDC: 1817210-1817211):

Compound	**2**	**3**
Colour, Habit	Light Brown, Plate	Green, Block
Size/mm	0.08 x 0.05 x 0.02	0.06 x 0.03 x 0.03
Empirical Formula	$C_{113}H_{185}O_6Si_8Ti_4$	$C_{55}H_{92}O_2Si_4Ti_2$
M	2055.87	993.44
Crystal System	Monoclinic	Monoclinic
Space Group	$P\ 2_1/n$	$P\ 2_1/n$
a/Å	14.5653(2)	12.9536(5)
b/Å	25.3232(5)	22.7968(9)
c/Å	31.1528(4)	18.9201(8)
α/°	90	90
β/°	91.2490(10)	92.537(4)
γ /°	90	90
V/ Å^3	11501.3(3)	5581.6(4)
Z	4	4
μ/mm^{-1}	3.460	0.410

T (K)	100	100
$\theta_{min/max}$	2.838/67.627	2.086/25.028
Completeness	98.9 to 67.073	99.9 to 25.028
Reflections: Total/Independent	20424/16144	9851/6048
R_{int}	0.0544	0.0976
Final *R1* and *wR2*	0.1285/0.3566	0.0836/0.2501
Goof	0.942	1.033
Largest peak hole/ e.Å^{-3}	2.784/-1.472	1.5 and -1.0
ρ_{calc}/g.cm^{-3}	1.189	1.182

Table S1

NMR SPECTRA OF COMPOUNDS (2) AND (3):

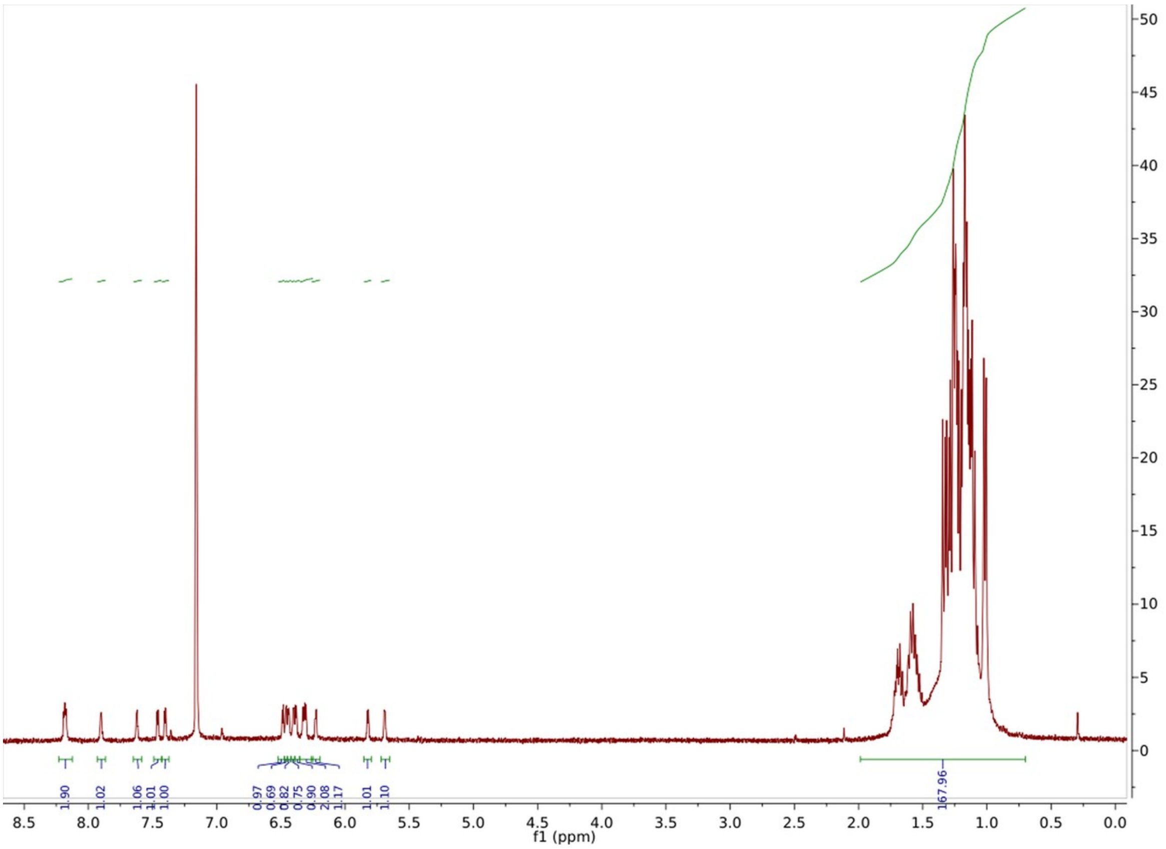

Figure S9: ^{1}H-NMR spectrum of (**2**) in C_6D_6.

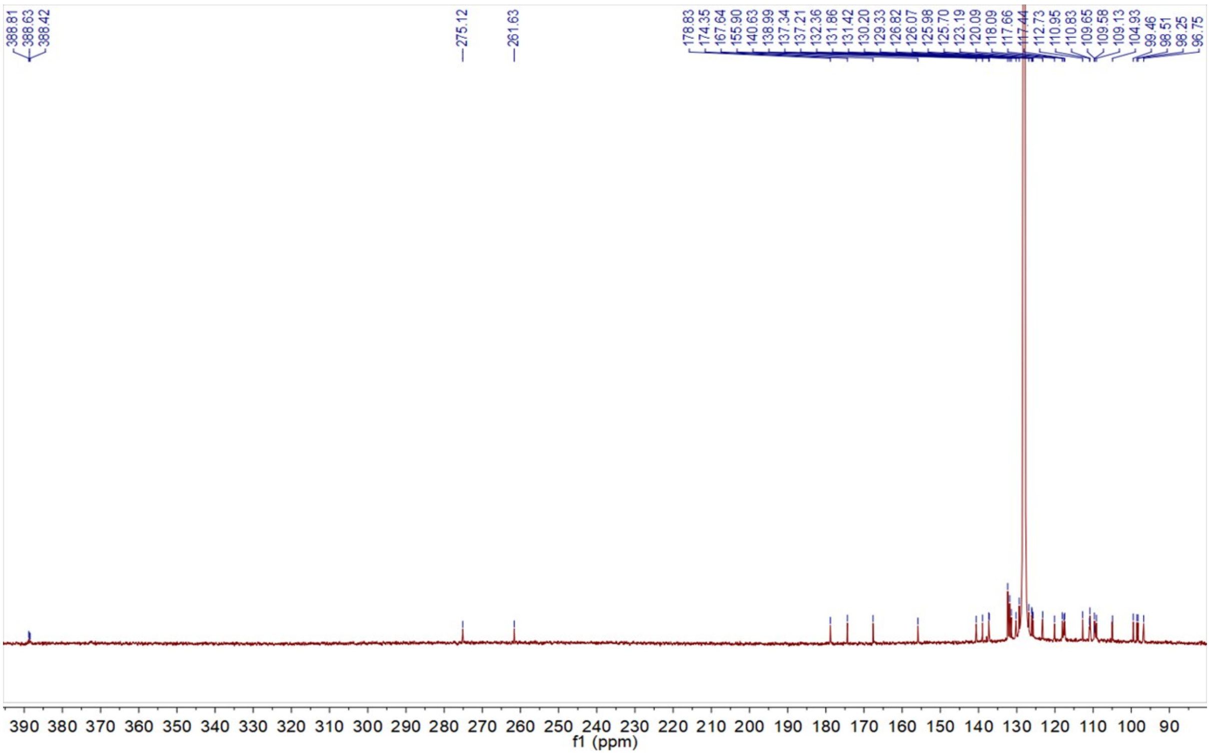

Figure S10: $^{13}C\{^{1}H\}$-NMR spectrum of (**2**) in C_6D_6.

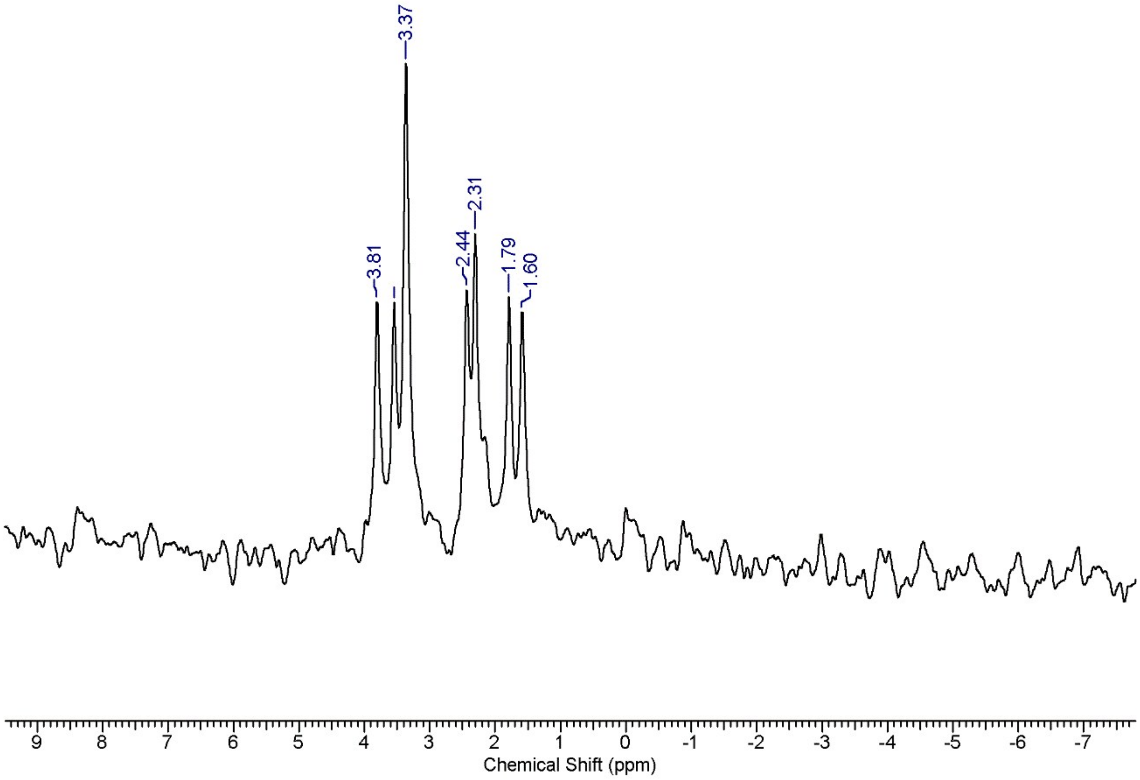

Figure S11: $^{29}Si\{^{1}H\}$-NMR spectrum of (**2**) in C_6D_6.

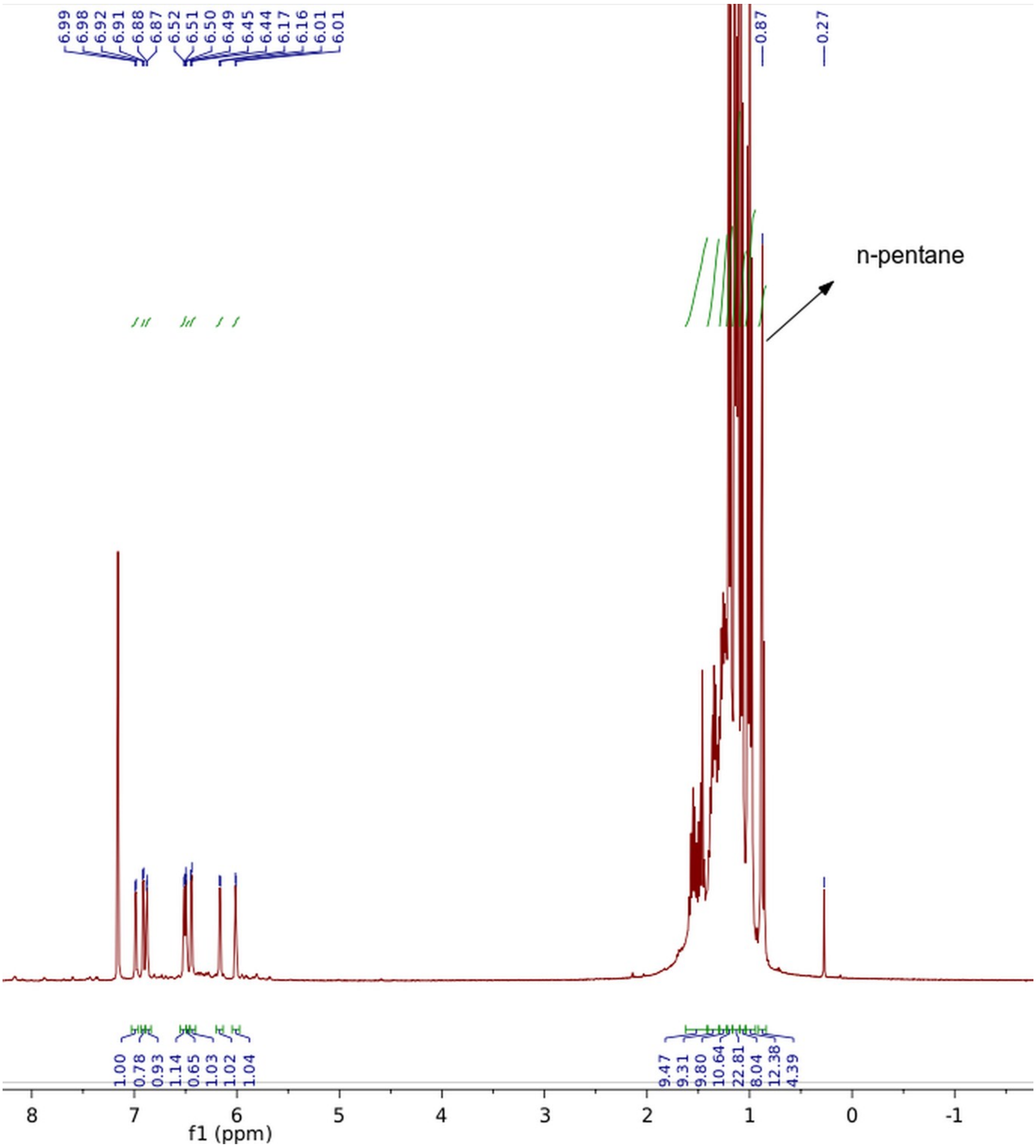

Figure S12: ^{1}H-NMR spectrum of (**3**) in C_6D_6.

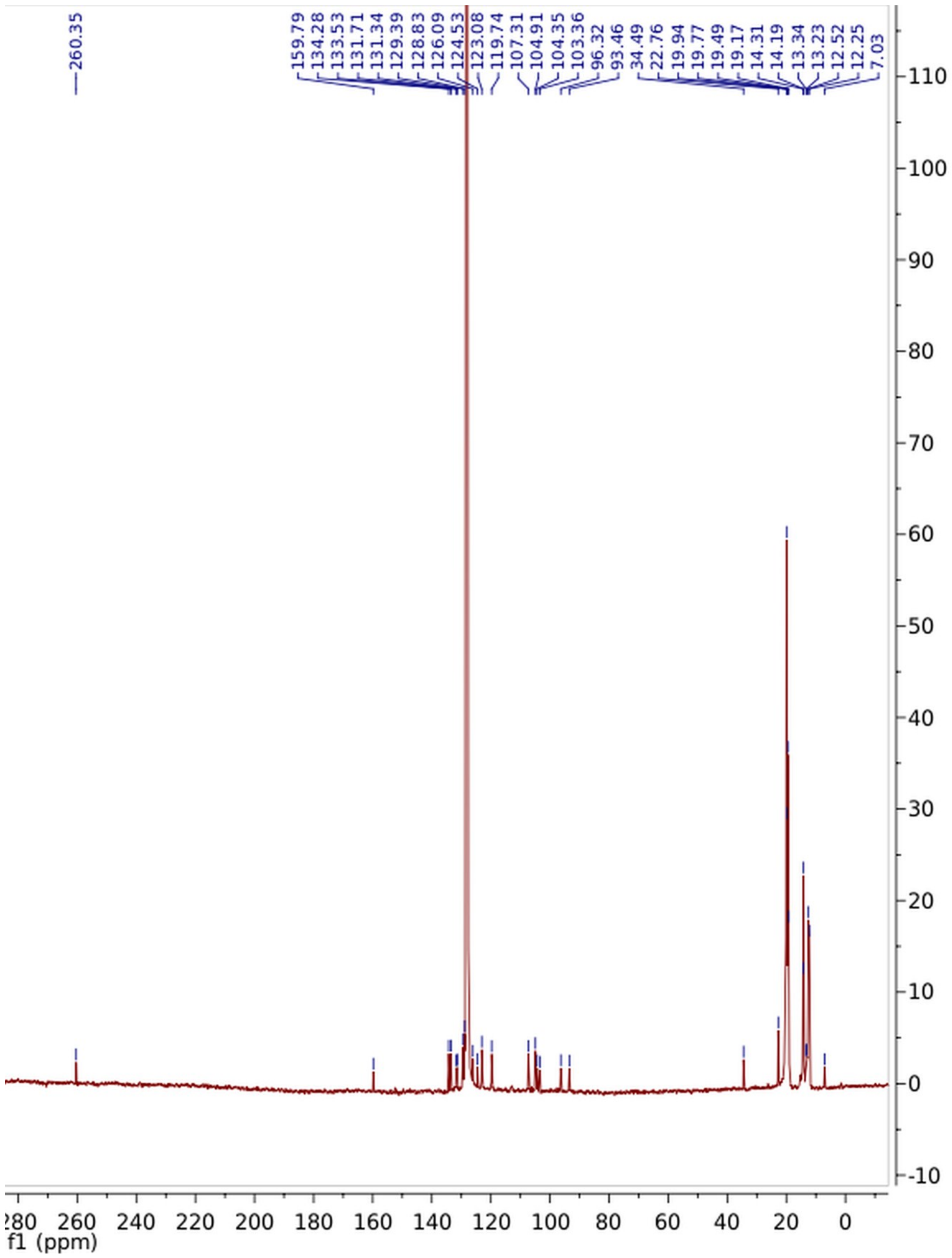

Figure S13: $^{13}C\{^{1}H\}$-NMR spectrum of (**3**) in C_6D_6.

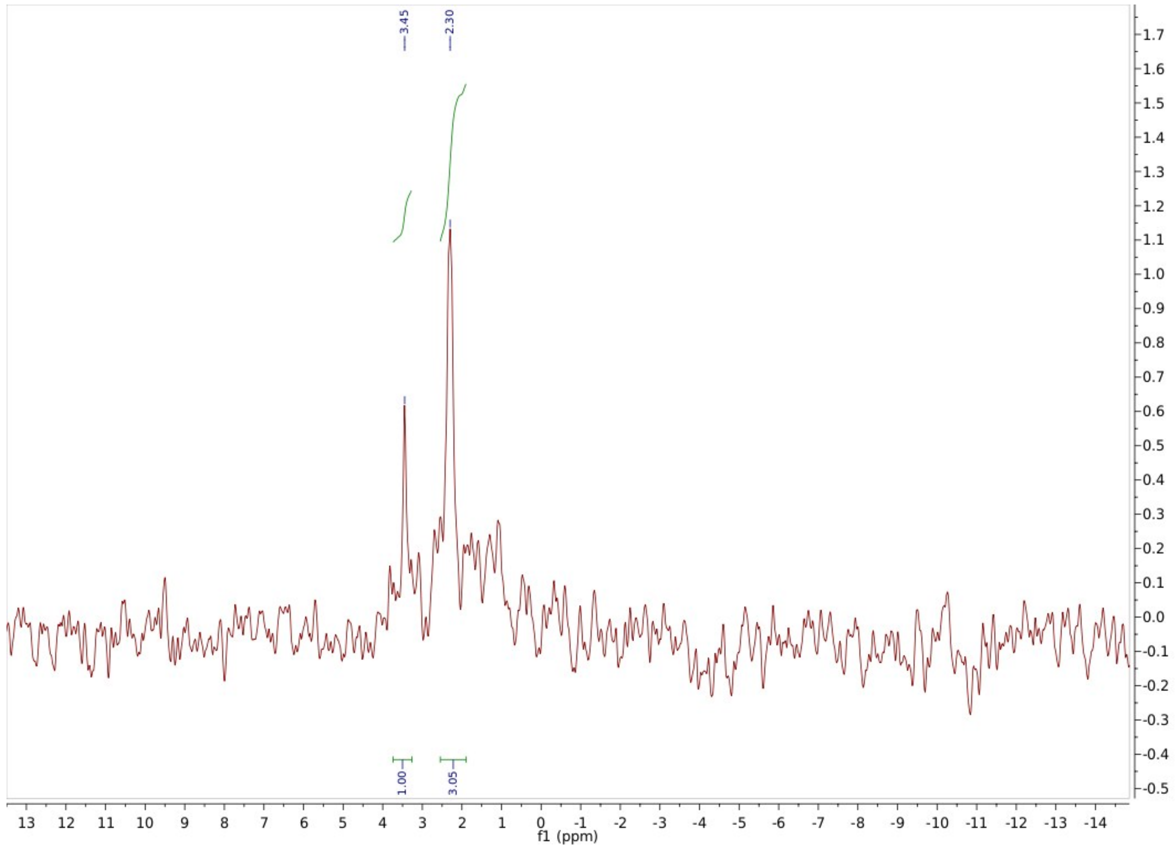

Figure S14: $^{29}Si\{^{1}H\}$-NMR spectrum of (**3**) in C_6D_6.

IR SPECTRA OF (1), (2) AND (3):

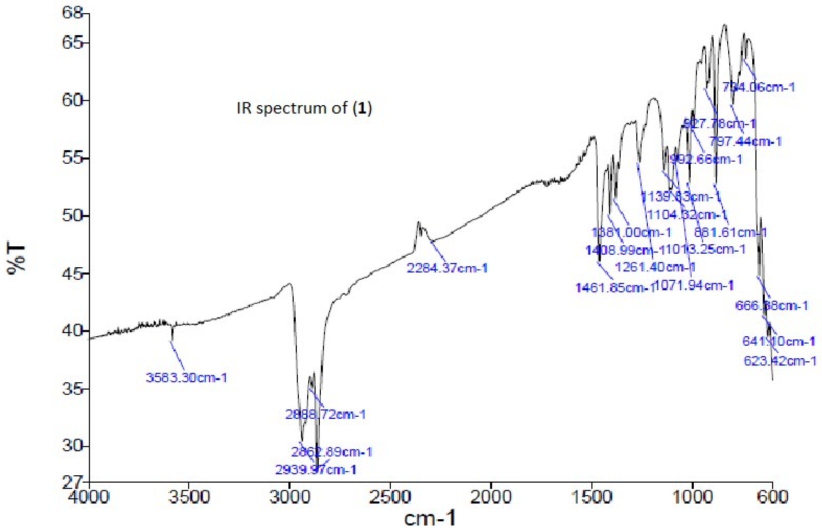

Figure S15: IR spectrum of (**1**).

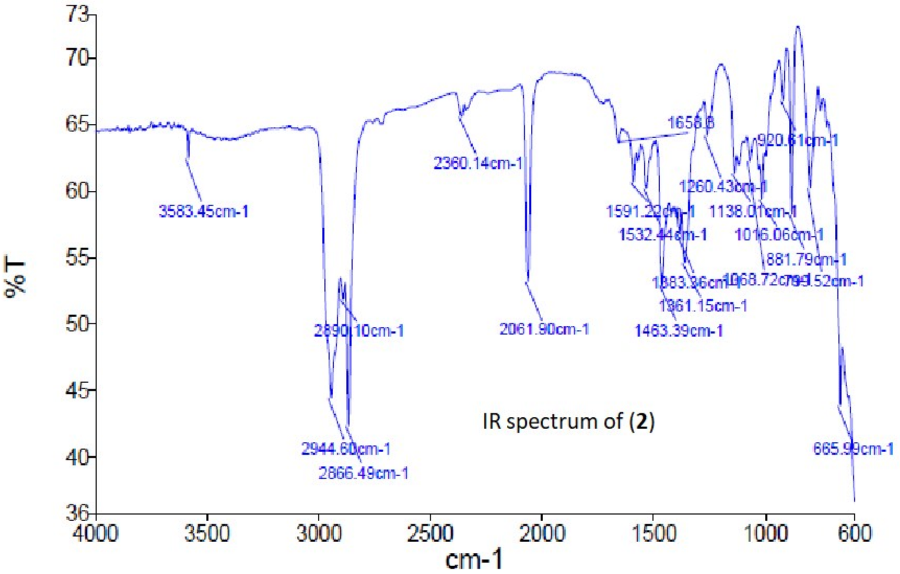

Figure S16: IR spectrum of (**2**).

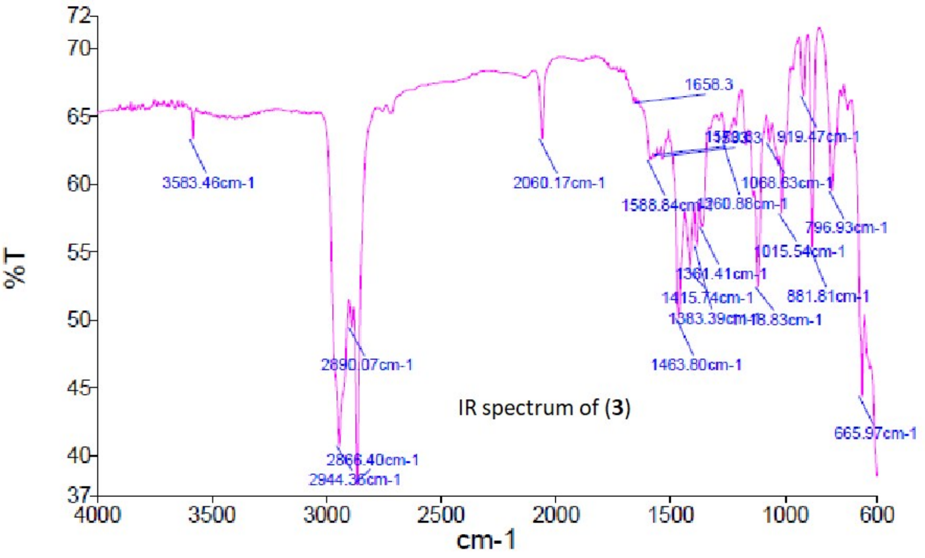

Figure S17: IR spectrum of (**3**).

Computational Details:

Density functional calculations were carried using the Amsterdam Density Functional package (version ADF2016.107).[6] The Slater-type orbital (STO) basis sets were of triple-ζ quality augmented with a one polarisation function (ADF basis TZP). Core electrons were frozen (C, O 1s; Ti 2p) in the model of the electronic configuration for each atom. The local density approximation (LDA) by Vosko, Wilk and Nusair (VWN)[7] was used together with the exchange correlation corrections of Becke and Perdew (BP86).[8,9] Local minima and transition states were confirmed by frequency calculations.

Table S2. Selected bond lengths (Å) for calculated structures ***1- 5***

	1	***3***	***3'***	***4***	***5***	***2***
Ti1-Ti2	2.37	2.41	2.41	2.48	2.45	2.45
Ti2-C1		2.17	2.10	2.27	2.25	2.24
Ti1-C1		2.21		2.42	2.49	2.53
Ti2-C2			2.17			
Ti1-C2		2.32	2.23			
Ti2-O1		2.19		2.06	2.04	2.04
C1-C2		1.37	1.57	1.44	1.44	1.43
C2-C6		1.30	1.30	1.34	1.47	1.49
C1-O1		1.26	1.20	1.28	1.27	1.27
C6-O4		1.18	1.19	1.16	1.23	1.23
Ti1-C'				2.23	2.26	2.26
C'-C3				1.45	1.39	1.40
C2-C3				1.52	1.41	1.40
C'-C9A				1.31	1.31	1.31
C9A-O2A				1.18	1.18	1.18
C3-O3				1.24	1.38	1.39
C6-C5					1.51	1.48
C5-C4					1.46	1.41
C4-O3					1.41	1.38
C4-O6A					1.21	1.26
C5-C7					1.46	1.42
C7-O5					1.16	1.27
Ti3-Ti4						2.44
Ti3-C7						2.23
Ti3-O5						2.06
Ti4-C7						2.44
Ti4-O6A						2.11

Cartesian coordinates for optimised structures:

2

75

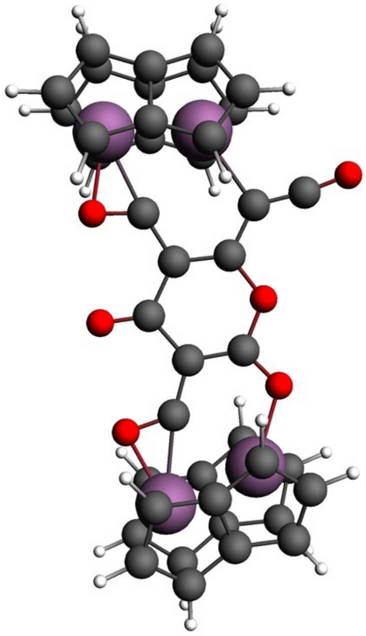

C	4.97957737	-1.23693438	1.86575855
C	4.84917220	-1.26640722	0.41439420
C	4.84695422	0.09332865	-0.05410024
C	5.06393402	0.92817736	1.07458823
C	5.07647082	0.14442042	2.26689414
C	4.80329189	-2.64668008	0.00805477
C	5.03783853	-2.60623470	2.32898537
C	5.02218762	-3.43830202	1.17006339
H	4.73670001	0.42887098	-1.08043964
H	5.16512949	2.01135838	1.03721497
H	5.26456193	0.51958563	3.26647952
H	4.71301033	-3.02000722	-1.00662313
H	5.23937534	-2.93832692	3.34166547
H	5.11226950	-4.52290502	1.17721698
C	2.07197137	-1.14069739	3.36299725
C	0.96456197	-1.13690381	2.41538286
C	0.65673906	0.23341793	2.10540741
C	1.49400683	1.04671074	2.91510767
C	2.41642642	0.23192882	3.63753235
C	0.58543390	-2.50578321	2.18119167
C	2.34180037	-2.51694593	3.71754953
C	1.36240372	-3.31627025	3.05551234
Ti	2.87542429	-2.48930311	1.43844355
H	1.46233517	2.13441718	2.94708569
H	3.14086723	0.58859123	4.36123389
H	-0.21036750	-2.85519135	1.53168614
H	3.03009033	-2.86535247	4.48022156

H	1.25800867	-4.39426474	3.16308793
Ti	2.91261738	-0.04152369	1.35017874
H	-0.09974461	0.59119519	1.41403220
C	2.00076270	-3.80334072	-0.18457489
C	1.97188174	-1.03801824	-0.42037475
O	1.99744710	0.23408987	-0.45137602
C	2.11527935	-5.07028658	0.11943206
O	2.28697661	-6.16586502	0.52825941
C	1.44858286	-3.20336310	-1.31848097
C	1.40759645	-1.81220568	-1.48248098
C	0.80553033	-1.19190332	-2.69734287
O	0.94470748	-4.06268149	-2.28044049
O	0.76373552	0.02509268	-2.88563541
C	0.36523818	-3.56587373	-3.43163456
C	0.26383444	-2.17854779	-3.66338211
C	-0.37741358	-1.82148789	-4.88184706
O	-0.57470335	-0.62472732	-5.26338174
O	-0.04505458	-4.46826686	-4.21493442
C	-3.22364889	-3.23847355	-6.83981937
C	-3.15507480	-2.69857429	-5.48751679
C	-3.37749574	-1.28137845	-5.56612984
C	-3.66817958	-0.97484503	-6.92278813
C	-3.51378974	-2.14255611	-7.73046013
C	-2.90946488	-3.79954013	-4.59226692
C	-3.04441868	-4.67344809	-6.75032116
C	-2.94972565	-4.99279545	-5.36074857
H	-3.36250239	-0.57322168	-4.74352308
H	-3.93706309	0.01204095	-7.29433403
H	-3.71477996	-2.20440794	-8.79397540
H	-2.79205791	-3.74454126	-3.51445580
H	-3.15383018	-5.38998022	-7.55743494

C	-0.29949906	-3.23708188	-8.33096972
C	0.75386570	-2.69369579	-7.48299946
C	0.81757597	-1.27695900	-7.71127435
C	-0.11121081	-0.97408411	-8.74333217
C	-0.85286664	-2.14421825	-9.09050697
C	1.33610766	-3.79221706	-6.75612032
C	-0.32807318	-4.67217575	-8.13208947
C	0.74184970	-4.98790915	-7.23959839
Ti	-0.96795387	-3.89728278	-6.02815531
H	-0.25710756	0.01208561	-9.17984196
H	-1.59654408	-2.20973993	-9.87668088
H	2.13998051	-3.73312283	-6.02876933
H	-0.91468730	-5.39115601	-8.69404437
H	1.01873640	-5.99161839	-6.92227453
Ti	-1.38893425	-1.64134515	-6.85689384
H	1.47191331	-0.56628114	-7.21633395
H	-2.85218640	-5.99719731	-4.95310045

3

35

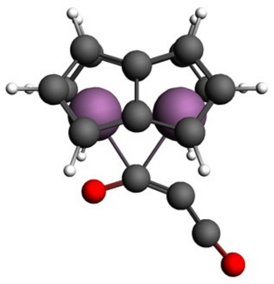

Ti	0.73079680	0.52143930	1.05569091
C	-1.73237595	0.34326904	1.34920155
C	-2.71144644	0.79297202	0.39019476
C	-2.85117208	-0.22690632	-0.58513350
H	-3.56364581	-0.20690156	-1.40789097

C	-1.88230692	-1.25479582	-0.36012278
H	-1.80277393	-2.17916487	-0.92164027
C	-1.20132047	-0.93044091	0.87594823
C	-0.26900378	-1.41152796	1.87339819
C	-0.29422535	-0.47408149	2.95426990
H	0.26370388	-0.57336106	3.88359283
C	-1.10695200	0.63619277	2.61319785
H	-1.25531011	1.52394999	3.22069283
C	2.18628260	-1.04585490	0.13962801
H	2.23221922	-2.07713665	0.47155511
C	3.01069987	0.02034383	0.61755343
H	3.71842035	-0.05598733	1.44103765
C	2.73046288	1.20967158	-0.10163631
C	1.81228976	0.86994578	-1.15890782
C	1.45591599	-0.53651160	-1.00144638
C	0.58524918	-0.89193673	-2.10230198
C	0.48086089	0.26178450	-2.94110417
H	-0.06332008	0.30394685	-3.88275758
C	1.14291344	1.36120664	-2.33753766
H	1.18999857	2.36987650	-2.73461915
C	-0.06829885	2.39669065	0.32050651
O	0.60641154	2.69291809	1.34668810
O	-1.86950573	4.99574457	-1.61815237
C	-1.34832232	4.06460306	-1.10915617
C	-0.83725428	2.96331545	-0.65752769
Ti	-0.66884785	0.65822324	-0.89976884
H	0.22020795	-1.88416544	-2.34382051
H	3.16628256	2.18560228	0.08980253
H	-3.28862606	1.71036344	0.43689827
H	0.23030242	-2.37390277	1.88330834

3’

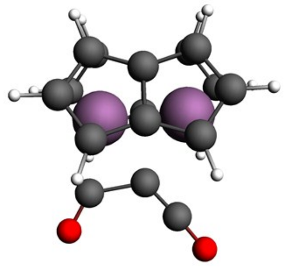

35

C	4.98152143	-1.28481566	1.85297323
C	4.97168115	-1.66459402	0.45123199
C	4.90122731	-0.46099532	-0.33682944
C	4.98979879	0.63304658	0.57234605
C	4.95733811	0.16084809	1.91397582
C	5.00130411	-3.10290073	0.38463467
C	5.06161977	-2.49632769	2.63737070
C	5.15614831	-3.58620286	1.71708310
H	4.91029141	-0.38789612	-1.41926072
H	5.02655009	1.68069176	0.27830745
H	5.03619353	0.77505491	2.80448906
H	5.00520036	-3.70824957	-0.51716561
H	5.18603271	-2.56673098	3.71229970
H	5.30208260	-4.62878321	1.99299107
C	1.89396345	-1.09674603	3.09456350
C	0.89059051	-1.42527973	2.09654124
C	0.51412864	-0.20748044	1.42524220
C	1.19158619	0.85605354	2.08864993
C	2.09791970	0.33511738	3.05412244
C	0.68072629	-2.84881784	2.13566454
C	2.26062669	-2.32127905	3.76967802
C	1.44845400	-3.36177771	3.22182541
Ti	2.99030084	-2.68942922	1.58416566

H	1.06495256	1.91142556	1.85344132
H	2.71580507	0.92342919	3.72415822
H	-0.01074240	-3.41766886	1.52048771
H	2.91295351	-2.41739464	4.63070473
H	1.43244025	-4.39332921	3.56827990
Ti	2.85980871	-0.37667407	0.91810512
H	-0.23243634	-0.09638457	0.64605004
C	2.23720343	-1.99302852	-0.39592787
C	2.07397436	-0.56028934	-1.02147846
O	1.66469385	-0.25241631	-2.11009841
C	1.91720112	-3.07897635	-1.03257813
O	1.60513506	-4.03604570	-1.67096808

4

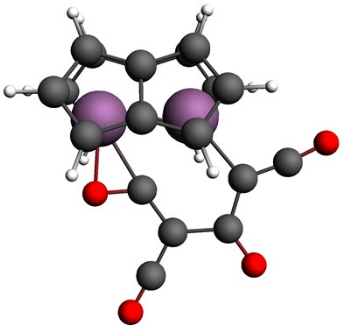

40

C	4.91031010	-1.29989065	1.76028128
C	4.90706667	-1.27004786	0.30279211
C	4.96840471	0.10585922	-0.10785768
C	5.10728371	0.89362663	1.06919916

C	5.00398928	0.06212058	2.22361554
C	4.86582571	-2.63054930	-0.16251918
C	4.89733240	-2.68544730	2.17088743
C	4.96108822	-3.46931773	0.98018158
H	4.97051769	0.48200857	-1.12632485
H	5.24402987	1.97320822	1.08543640
H	5.11859511	0.39257416	3.24961162
H	4.84706780	-2.96685090	-1.19387669
H	5.00802448	-3.06245298	3.18204477
H	5.01634278	-4.55590648	0.94997807
C	1.89124207	-1.13608562	3.01795198
C	0.86237227	-1.05918916	1.98886515
C	0.64756382	0.32970103	1.68567406
C	1.45166512	1.08770564	2.58228230
C	2.27148374	0.21293679	3.35585201
C	0.44132014	-2.40182158	1.69042810
C	2.07263673	-2.52967665	3.35504855
C	1.11312145	-3.26518875	2.59686127
Ti	2.80108108	-2.49933141	1.11370849
H	1.46015839	2.17410917	2.64696243
H	2.94571857	0.51809524	4.14792922
H	-0.30831611	-2.70453762	0.96679962
H	2.68214224	-2.92747898	4.15940589
H	0.95798310	-4.34061454	2.66007127
Ti	2.93199056	-0.02473952	1.11588040
H	-0.04251737	0.73783190	0.95366054
C	2.04950798	-3.84273738	-0.50118013
C	2.10763539	-1.08509165	-0.71919706
O	2.15567268	0.19152943	-0.78270677
C	2.16209760	-5.09317522	-0.13547516
O	2.32420905	-6.17418888	0.31913263

C	1.51763070	-3.37568376	-1.76291056
C	1.58912677	-1.86285289	-1.82038009
C	1.16486850	-1.21041381	-2.91521924
O	1.05881384	-4.03325670	-2.70193500
O	0.81124585	-0.60400043	-3.83993614

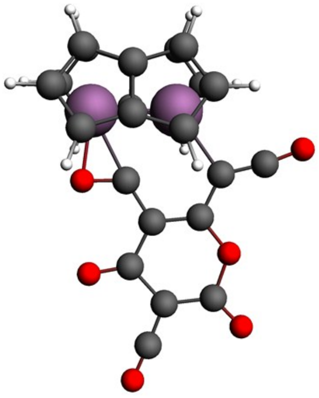

5

45

C	4.96708762	-1.32250468	1.45680952
C	4.72212326	-1.24395048	0.02230454
C	4.73687592	0.14472837	-0.35046585
C	5.07751431	0.88908275	0.81131549
C	5.15218781	0.02244780	1.94189294
C	4.58658288	-2.58973900	-0.47179989
C	5.00698561	-2.72235155	1.81968759
C	4.86568350	-3.46854262	0.61187196
H	4.56656307	0.55553956	-1.34061041
H	5.22228710	1.96754130	0.83931999
H	5.43376669	0.31844055	2.94625064
H	4.40715495	-2.88747669	-1.49971723
H	5.27700695	-3.13245779	2.78682056
H	4.91506727	-4.55318787	0.53693553

C	2.19306215	-1.21671425	3.19051605
C	1.01720810	-1.10684349	2.33694308
C	0.74896192	0.29200465	2.13632463
C	1.68178800	1.01658443	2.92682558
C	2.61820033	0.12025668	3.52184473
C	0.55990861	-2.44126948	2.04975109
C	2.42917972	-2.62277760	3.43495695
C	1.36677772	-3.33738135	2.80541484
Ti	2.78619662	-2.46923065	1.12666930
H	1.70086874	2.09999090	3.03032974
H	3.41225297	0.39890331	4.20538531
H	-0.30070720	-2.71602897	1.44865748
H	3.15920833	-3.04814780	4.11516716
H	1.22107271	-4.41478415	2.85589022
Ti	2.91830746	-0.02205915	1.18818988
H	-0.04412235	0.72419465	1.53442663
C	1.73395295	-3.66886264	-0.47826482
C	1.80768330	-0.90746342	-0.55272325
O	1.87314229	0.36342630	-0.52333079
C	1.80406227	-4.95812699	-0.24792843
O	1.94925864	-6.07668405	0.09303248
C	1.12358483	-3.00534380	-1.54100660
C	1.13920659	-1.59862481	-1.61803123
C	0.51145818	-0.88657054	-2.73748563
O	0.52066122	-3.80190113	-2.49254286
O	0.47901267	0.33115696	-2.90309663
C	-0.12195074	-3.28316151	-3.63198488
C	-0.12150353	-1.82876288	-3.73049228
C	-0.73194286	-1.28922916	-4.79622070
O	-1.24612615	-0.77306336	-5.69690986
O	-0.60600389	-4.07340682	-4.41289743

Transition state **3** + C_3O_2 = **4**

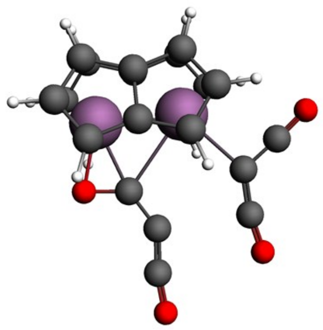

40

Ti	0.56399600	0.77487800	0.97727500
C	-1.82544100	0.07449900	1.11827300
C	-2.74506700	0.09114000	0.01216600
C	-2.56845000	-1.12977900	-0.70230300
H	-3.18070100	-1.46490100	-1.53676200
C	-1.43853500	-1.83299900	-0.18791700
H	-1.11009600	-2.81591800	-0.50713800
C	-0.99633900	-1.11755700	0.98970900
C	-0.11890400	-1.16789100	2.13109100
C	-0.46495200	-0.06137900	2.96497800
H	-0.00716100	0.16111000	3.92759000
C	-1.44318300	0.74397500	2.32967500
H	-1.83985000	1.68092200	2.70703300
C	2.39033800	-0.64774100	0.52264000
H	2.59363400	-1.54535100	1.09616600
C	2.94049300	0.64554900	0.77677800
H	3.55479200	0.90392200	1.63791900
C	2.51615200	1.56259000	-0.21733200

C	1.78133100	0.81653300	-1.19999300
C	1.69753000	-0.56379500	-0.73812300
C	1.02671400	-1.32912200	-1.76670800
C	0.79432300	-0.44296700	-2.86035300
H	0.38075800	-0.73920100	-3.82208000
C	1.14495600	0.88804300	-2.48810900
H	1.04289600	1.77379200	-3.10684000
C	-0.36870400	2.54886500	0.10196400
O	0.29285400	2.75907800	1.19674900
O	-1.72302700	5.83077400	-0.93380600
C	-1.33968800	4.73034000	-0.69648800
C	-1.03722900	3.47659700	-0.63306300
Ti	-0.64021900	0.15371400	-1.09761100
H	0.89941600	-2.40572500	-1.79102900
H	2.73003900	2.62637000	-0.23565700
H	-3.50107700	0.84449400	-0.18422700
H	0.56536500	-1.96757100	2.39183300
O	-2.70821300	3.71158900	-3.31647900
C	-2.29360400	2.69170700	-2.93014500
C	-1.91873900	1.43297800	-2.69210400
C	-2.23117700	0.41526400	-3.46681200
O	-2.49040400	-0.52197000	-4.12825400

Transition state **4** + C_3O_2 = **5**

45

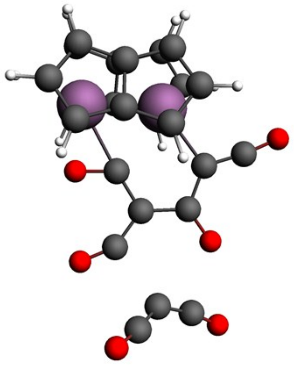

C	5.03372916	-1.07750925	1.78771442
C	4.94512330	-1.10711234	0.33332799
C	4.82512515	0.24924276	-0.12749168
C	4.93955024	1.09376254	1.01299760
C	4.99759079	0.30346036	2.19833981
C	5.03451689	-2.48070720	-0.08271003
C	5.20100648	-2.43821410	2.24500243
C	5.28994470	-3.25663797	1.07999999
H	4.72520863	0.58126408	-1.15615747
H	4.95159842	2.18181645	0.98590389
H	5.12791389	0.68563550	3.20436367
H	4.99960590	-2.85701987	-1.09963000
H	5.40489154	-2.76029385	3.26067587
H	5.46816750	-4.33038656	1.08355552
C	2.08406500	-1.19502830	3.21040715
C	1.00492624	-1.27834986	2.23494074
C	0.61925148	0.06378503	1.89156315
C	1.37510240	0.94447446	2.71558472
C	2.32499165	0.20102488	3.47709532
C	0.72582987	-2.67105661	2.00782412

C	2.43873586	-2.54423529	3.58818694
C	1.53434645	-3.41387879	2.91076469
Ti	3.04137975	-2.53269638	1.30307134
H	1.26474009	2.02702387	2.73843211
H	3.00102257	0.61493569	4.21655247
H	-0.02016075	-3.08645549	1.33875998
H	3.13116406	-2.83644776	4.37066454
H	1.50651844	-4.49611527	3.02285872
Ti	2.88934938	-0.05747355	1.20792495
H	-0.14812026	0.35892398	1.18256562
C	2.37944479	-4.02659575	-0.20584912
C	2.08737030	-1.32551604	-0.54122533
O	2.00259800	-0.05458127	-0.64077203
C	2.67948552	-5.23894481	0.18303616
O	3.00972439	-6.27039434	0.65967783
C	1.72969685	-3.66518539	-1.44065249
C	1.56173147	-2.19885164	-1.56896792
C	0.96188183	-1.50935267	-2.59869824
O	1.37504508	-4.48073143	-2.32415653
O	0.75979127	-0.47828743	-3.12849361
C	-0.48576783	-2.95333265	-3.62747528
C	-0.00921957	-4.07717861	-4.12617871
O	0.31468484	-5.01064678	-4.74692362
C	-1.64268502	-2.36300461	-3.67137548
O	-2.63739701	-1.74278852	-3.61986083

Transition state **4** + **3** = **2**

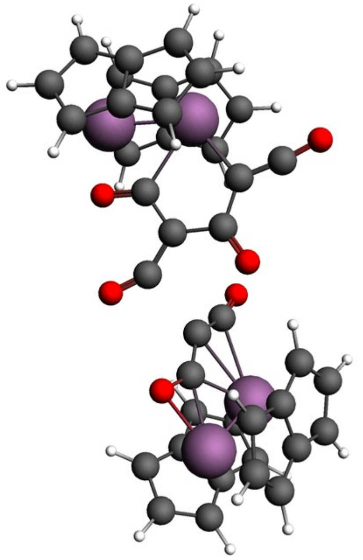

C	5.26678932	-1.59068629	0.86010532
C	4.63448444	-1.25443480	-0.40896315
C	4.64961901	0.17725896	-0.54147766
C	5.36309838	0.69740140	0.57466517
C	5.68762341	-0.35867022	1.47766509
C	4.25971341	-2.48454395	-1.05262555
C	5.29255389	-3.03164245	0.96892987
C	4.75560460	-3.54594405	-0.24940386
H	4.24468792	0.75849075	-1.36386410
H	5.59993497	1.74833319	0.73010196
H	6.26473062	-0.25375991	2.38939312
H	3.75733107	-2.59155355	-2.00831233
H	5.78695028	-3.61523724	1.73824099
H	4.67697029	-4.60229144	-0.49966914
C	3.12083744	-1.66420219	3.33977770
C	1.76200317	-1.36887492	2.90478121
C	1.57408253	0.05426198	2.98599596
C	2.75765700	0.60483791	3.55292100

C	3.73990763	-0.41766002	3.71311790
C	1.12350972	-2.61698256	2.58416612
C	3.29336183	-3.09833847	3.29717348
C	2.03366302	-3.65397673	2.92269343
Ti	2.97313412	-2.60902551	1.00808031
H	2.90214789	1.65504075	3.79968427
H	4.71908503	-0.28582001	4.15930782
H	0.11030094	-2.75699136	2.22309252
H	4.15056294	-3.65891620	3.65468797
H	1.81909025	-4.71812815	2.84461479
Ti	3.33714325	-0.19003284	1.40601164
H	0.68173699	0.60948641	2.71490018
C	1.47585756	-3.57340023	-0.31536104
C	1.69228862	-0.83811278	-0.05014448
O	1.85268039	0.41866954	0.13204877
C	1.59671035	-4.87025249	-0.23718948
O	1.82586815	-6.02410082	-0.08203412
C	0.50916682	-2.83020455	-1.09863529
C	0.71305796	-1.36196265	-0.96277761
C	0.01909904	-0.37136893	-1.63429922
O	-0.41033706	-3.31767426	-1.78054547
O	-0.28208765	0.76399434	-1.69433073
Ti	-3.66013428	-0.94683577	-5.30506346
C	-2.03701814	0.44133784	-6.57779450
C	-0.62488256	0.17097150	-6.66445565
C	-0.43767505	-0.80043909	-7.68268962
H	0.52937271	-1.16631942	-8.02271312
C	-1.70446747	-1.26849338	-8.14687667
H	-1.85899978	-1.97487630	-8.95503326
C	-2.71696036	-0.46560815	-7.49478450
C	-4.12808795	-0.15361412	-7.45036481

C	-4.28116635	0.96159881	-6.56948877
H	-5.22223583	1.46677597	-6.35920162
C	-3.03783445	1.27743847	-5.96441035
H	-2.87123240	2.04435014	-5.21435874
C	-4.92668090	-2.72845966	-6.12337432
H	-5.54413881	-2.66303716	-7.01228379
C	-5.37147522	-2.49821014	-4.78325593
H	-6.36161372	-2.13953853	-4.50728738
C	-4.31525202	-2.74927715	-3.87130754
C	-3.21428077	-3.29050391	-4.62767424
C	-3.58854861	-3.26869189	-6.03637587
C	-2.49761051	-3.85688996	-6.78322274
C	-1.52698935	-4.29580428	-5.82472497
H	-0.60895268	-4.82910749	-6.06483654
C	-1.90276978	-3.87933576	-4.52322208
H	-1.32892437	-4.00241481	-3.60664898
C	-1.87801602	-0.83165107	-4.01354870
O	-2.84365998	-0.24937403	-3.44099253
O	1.61668275	-1.94193657	-4.50169137
C	0.49591142	-1.59591981	-4.28217132
C	-0.56969959	-1.09323994	-3.70629860
Ti	-1.54322215	-1.93250748	-5.90978882
H	-2.48951133	-4.08982920	-7.84259322
H	-4.35118405	-2.60876406	-2.79549726
H	0.16386013	0.67176042	-6.11162996
H	-4.90553562	-0.57268401	-8.07901840

References:

[1] Kilpatrick, A. F. R., Green, J. C., Cloke, F. G. N. & Tsoureas, N. *Chem. Commun.* **49,** 9434-9436 (2013).

[2] Hillhouse, G. L. *J. Am. Chem. Soc.***107,** 7772-7773 (1985)

[3] Coles, S. J., Gale, P. A. *Chem. Sci.* **3,** 683-689 (2012).

[4] Farrugia, L. J. *J. Appl. Cryst.* **32,** 837-838 (1999).

[5] Dolomanov, O. V., Bouhris, L. J., Gildea, R. J., Howard, J. A. K. and Puschmann, H. *J. Appl. Cryst.* **42,** 339-341 (2009).

[6] SCM, *Amsterdam Density Funstional (ADF)*, Theoretical Chemistry, Vrije Universiteit, Amsterdam, The Netherlands, 2016.

[7] Vosko, S. H., Wilk, L. and Nusair, M. *Can. J. Phys.*, **58**, 1200–1211 (1980).

[8] Becke, A. *Phys. Rev., A*, **38**, 3098–3100 (1988).

[9] Perdew, J. P. *Phys. Rev. B*, **33**, 8822–8824 (1986).
